# Haplotype based testing for a better understanding of the selective architecture

**DOI:** 10.1186/s12859-023-05437-3

**Published:** 2023-08-26

**Authors:** Haoyu Chen, Marta Pelizzola, Andreas Futschik

**Affiliations:** 1 University of Veterinary Medicine Vienna, Vienna, Austria; 2 Vienna Graduate School of Population Genetics, Vienna, Austria; 3 Aarhus University, Aarhus, Denmark; 4 Johannes Kepler University of Linz, Linz, Austria

**Keywords:** Hypothesis test, Post hoc test, Selection, Evolve and resequence, Experimental evolution, Haplotype

## Abstract

**Background:**

The identification of genomic regions affected by selection is one of the most important goals in population genetics. If temporal data are available, allele frequency changes at SNP positions are often used for this purpose. Here we provide a new testing approach that uses haplotype frequencies instead of allele frequencies.

**Results:**

Using simulated data, we show that compared to SNP based test, our approach has higher power, especially when the number of candidate haplotypes is small or moderate. To improve power when the number of haplotypes is large, we investigate methods to combine them with a moderate number of haplotype subsets. Haplotype frequencies can often be recovered with less noise than SNP frequencies, especially under pool sequencing, giving our test an additional advantage. Furthermore, spurious outlier SNPs may lead to false positives, a problem usually not encountered when working with haplotypes. Post hoc tests for the number of selected haplotypes and for differences between their selection coefficients are also provided for a better understanding of the underlying selection dynamics. An application on a real data set further illustrates the performance benefits.

**Conclusions:**

Due to less multiple testing correction and noise reduction, haplotype based testing is able to outperform SNP based tests in terms of power in most scenarios.

**Supplementary Information:**

The online version contains supplementary material available at 10.1186/s12859-023-05437-3.

## Background

Evolve and Resequence (E &R) experiments [1] provide a modern approach for studying patterns of adaptation in a controlled environment. In such experiments, one or multiple populations are followed over time, often under stressful environmental conditions. Researchers then aim to identify adaptive changes at a genetic level. High-throughput whole genome sequencing techniques provide allele and haplotype frequency data at suitable time points during the experiment. Depending on the experimental design and the available resources, sequencing can be performed at the beginning and the end of the experiment or at multiple time points. The experiment is often also replicated so that analogous data are obtained from multiple populations.

Once data are available, statistical tests are often used to identify the presence of selection. A proper test needs to take all sources of random variation into account. Indeed, besides selection, observed allele frequencies are affected by genetic drift, and frequently also by sampling and sequencing noise. So far, different SNP based tests for selection have been proposed in the context of E &R experiments. Some approaches, such as [2] are heuristic and do not control the type I error. More recently, in [3] a modified version of the classical chi-square and CMH test has been proposed that is able to take all relevant sources of randomness into account. For a review of further available methods, we refer to [4].

Here we propose tests that rely on haplotype frequencies instead of SNP frequencies and illustrate their potential and advantages. We define haplotypes as alleles that are defined by their unique combination of SNP genotypes on some window of predefined size and location. Notice that the term “haplotype” is also used to refer to the locus itself instead of an allele. In this manuscript, we will mostly use the allelic definition, with the meaning being apparent from the context. If we state, for instance, that at a chosen locus, the relative frequencies of $N_{\text {Hap}}$ haplotypes add up to one, this could also be phrased in terms of the corresponding alleles. However, since we use the notion “allele frequencies” for the nucleotides that appear at a SNP location, we use “haplotype frequencies” to avoid any confusion. Notice that the short loci we are focusing on are often called microhaplotypes [5].

In the different contexts of genome-wide association studies (GWAS), testing based on haplotypes has already been used [6, 7] to identify genetic variants that are associated with phenotypic traits of interest. Available methods include likelihood ratio tests [8] and score tests [9], and recently a combination of haplotype block and SNP set approaches have been proposed in [10].

For samples from natural populations, yet further examples of haplotype based tests can be found in [11] and its extension [12], where signatures of recent selection are found by searching for regions with long range linkage disequilibrium. The haplotype based test proposed in [13] is similar in spirit to [11] but focuses on ongoing selection.

Given that haplotype based testing has already proved promising in the above mentioned setups, it seems desirable to make it available also for E &R experiments. Since this involves temporal allele frequency data and a different null model, our proposed tests require a new methodological approach that is explained in detail in "Methods" section. In summary, we identify selected genomic windows by testing each haplotype against the combination of all others using a modification of the chi-square or (with replicate populations) the Cochran–Mantel–Haenszel (CMH) test [3]. The tests take all relevant sources of random variation into account. Subsequently, we combine the resulting *p* values using recently proposed combination tests for the global null hypothesis.

When the actual haplotype frequencies are not available, haplotype reconstruction techniques [14, 15] provide the possibility of estimating this information from allele frequency data. The standard error of these estimates will then be one of the sources of random variation.

In our simulations, we observed that haplotype based tests do not necessarily outperform SNP-based methods if the total number of haplotypes gets too large. Therefore we propose two variants of our approach in "Testing when many haplotypes are present" section that provide improved power with experiments involving many haplotypes.

Furthermore, when the presence of selection is established by the haplotype based test, we provide a post hoc test for the number of selected haplotypes in "Testing for the number of selected haplotypes" section. In "Pairwise test for different fitness across haplotypes" section, we propose another post hoc test for differences in fitness between pairs of haplotypes. We provide results on simulated data for these two tests and show that they have good power under many scenarios ("Simulation experiments" section). In "Real data application" section, we also apply our proposed tests on real data considered by [16].

## Methods

Consider a haploid population with effective population size $N_e$, and a genomic region exhibiting $N_{{\text {Hap}}}$ haplotypes. We summarise the temporal dynamics of these haplotypes over $T+1$ generations via the relative haplotype frequencies $\mathbf {f_t}=(f_{1,t}, f_{2,t},\ldots ,f_{N_{{\text {Hap}}},t})^\intercal$ at generation *t* ($0\le t \le T$) where $\sum _{n=1}^{N_{{\text {Hap}}}} f_{n,t} = 1$. Without selection, the relative haplotype frequencies in a subsequent generation are obtained via multinomial sampling from the previous generation [17]:

1 $$\begin{aligned} \mathbf {f_{t+1}}\sim {\text {Multinomial}}(N_e, \mathbf {f_t})/N_e \end{aligned}$$

The changes in haplotype frequencies caused by repeated multinomial sampling are commonly known as genetic drift. Under selection, the haplotypes differ in fitness, leading to modified multinomial sampling probabilities [18, 19]:

2 $$\begin{aligned} \mathbf {f_{t+1}}\sim {\text {Multinomial}}(N_e, C_t\mathbf {f_t}\odot \Phi )/N_e \end{aligned}$$

where

3 $$\begin{aligned} \Phi =(\phi _1,\phi _2,\ldots ,\phi _{N_{{\text {Hap}}}})^\intercal \end{aligned}$$

is the fitness vector, $C_t$ a normalising constant at time *t* such that $C_{t}\sum _{j=1}^{N_{{\text {Hap}}}}f_{j,t} \phi _{j}=1$, and $\odot$ the element-wise multiplication defined as

$$\begin{aligned} (f_{1,t},f_{2,t},\ldots ,f_{N_{{\text {Hap}}}})^\intercal \odot (\phi _1,\phi _2,\ldots ,\phi _{N_{{\text {Hap}}}})^\intercal =(f_{1,t}\phi _1,f_{2,t}\phi _2,\ldots ,f_{N_{{\text {Hap}}}}\phi _{N_{{\text {Hap}}}})^\intercal \end{aligned}$$

Neutrality then corresponds to the special case where all elements of $\Phi$ are equal and $C_t\phi _1=C_t\phi _2=\cdots =C_t\phi _{N_{{\text {Hap}}}} =1$. Otherwise, higher fitness of a certain haplotype compared to others represents the presence of a selective advantage.

In the context of evolve and resequence, the experiment is often replicated, which leads to R independent haplotype frequency vectors $\mathbf {f_t}^{(1)},\,\mathbf {f_t}^{(2)},\ldots , \mathbf {f_t}^{(R)}$ at any sequenced time point *t*. Suppose we have an experiment with *k* sequenced time points $(t_0,t_1,\ldots ,t_{k-2},t_{k-1})$, with $t_0 = 0$ and $t_{k-1}=T$. Table 1 displays the haplotype frequency matrix $\textbf{F}^{(r)}$, for some replicate population *r*. These matrices may then be combined in to a $N_{{\text {Hap}}}\times kR$ matrix $$\textbf{F}=\begin{bmatrix}\textbf{F}^{(1)}&\textbf{F}^{(2)}&\ldots&\textbf{F}^{(R)}\end{bmatrix}$$. If the true frequencies are unknown, we use estimates $\hat{\mathbf {f}}^{(r)}_t =(\hat{f}^{(r)}_{1,t},\hat{f}^{(r)}_{2,t},\ldots ,\hat{f}^{(r)}_{N_{{\text {Hap}}},t})^\intercal$, and $$\hat{\textbf{F}}^{(r)} = \begin{bmatrix}\hat{\mathbf {f}}^{(r)}_0&\hat{\mathbf {f}}^{(r)}_{t_1}&\ldots& \hat{\mathbf{f}}^{(r)}_{t_{k-2}}& \hat{\mathbf{f}}^{(r)}_T\end{bmatrix}$$ instead of the actual quantities. These estimates will typically contain sampling and sequencing noise that needs to be taken into account when testing hypotheses.

**Table 1:** Structure of (true) haplotype frequency matrix with *k* sequenced time points for some replicate population *r*. The estimated frequency of haplotype *j* at generation *t* for replicate *r* is denoted by $\hat{f}_{j,t}^{(r)}$

Haplotype	Generation				
	0	$t_1$	$\ldots$	$t_{k-2}$	T
1	$f_{1,0}^{(r)}$	$f_{1,t_1}^{(r)}$	$\ldots$	$f_{1,t_{k-2}}^{(r)}$	$f_{1,T}^{(r)}$
2	$f_{2,0}^{(r)}$	$f_{2,t_1}^{(r)}$	$\ldots$	$f_{2,t_{k-2}}^{(r)}$	$f_{2,T}^{(r)}$
$\vdots$	$\vdots$	$\vdots$		$\vdots$	$\vdots$
$N_{{\text {Hap}}}$	$f_{N_{{\text {Hap}}},0}^{(r)}$	$f_{N_{{\text {Hap}}},t_1}^{(r)}$	$\ldots$	$f_{N_{{\text {Hap}}},t_{k-2}}^{(r)}$	$f_{N_{{\text {Hap}}},T}^{(r)}$
$\sum ^{N_{{\text {Hap}}}}_{j=1}f_{j,t}^{(r)}$	1	1	$\ldots$	1	1

To test for selection against the neutral null hypothesis $\phi _1=\phi _2=\cdots =\phi _{N_{{\text {Hap}}}} =\bar{\phi }$, where $\bar{\phi }=\frac{1}{N_{{\text {Hap}}}}\sum _{i=1}^{N_{{\text {Hap}}}} \phi _i$ is the mean fitness, we decompose the global null hypothesis into a multiple testing problem. For each haplotype *j* ($1\le j\le N_{{\text {Hap}}}$), distinguishing neutrality from selection may be phrased in terms of the hypothesis testing problem:

4 $$\begin{aligned} \begin{aligned} \mathbf {H_0}_j:\; \phi _j&=\bar{\phi } \\ \mathbf {H_1}_j:\; \phi _j&\ne \bar{\phi } \end{aligned} \end{aligned}$$

We propose the usage of the adapted CMH test [3] when multiple independent replicate populations are available. It naturally reduces to the adapted chi-square test if there exists only one replicate. The test is conducted in a binary fashion such that for some haplotype *j*, the test is conducted between the frequencies of haplotype *j* across all replicates $\{f_{j,\cdot }^{(r)}\}_{r\in \{1,2,\ldots ,R\}}$ and the cumulative frequencies of all other haplotypes $\{1-f_{j,\cdot }^{(r)}\}_{r\in \{1,2,\ldots ,R\}}$.

As discussed in [3] in the context of SNPs, the estimated haplotype frequencies $\hat{f}_{j,\cdot }^{(r)}$ may involve multiple components of variance. For simplicity, we present the test statistic assuming that all haplotype frequencies are known, and the only relevant variance component is genetic drift. For cases where other sources of variance such as sampling and pool sequencing noise are present, the test statistic can be found in Additional file 1: Section S.2 (the prefix S- refers to sections/figures/tables in the Additional file 1). For some haplotype *j*, the adapted CMH test using known haplotype frequencies has the following test statistic:

5 $$\begin{aligned} T_{{\text {CMH}}}=\frac{\sum ^R_{r=1}N_e^{{(r)}^4}\left( f_{j,0}^{(r)}-f_{j,T}^{(r)}\right) ^2}{\sum ^R_{r=1}N_e^{{(r)}^3}(N_e^{(r)}-1)\sigma _{{\text {drift}}}^{(r)}} \end{aligned}$$

where $\sigma _{{\text {drift}}}^{(r)}$ is the variance of haplotype frequencies due to drift at replicate *r*:

6 $$\begin{aligned} \sigma _{{\text {drift}}}^{(r)} =\sum ^{k-2}_{i=0}f_{j,t_i}^{(r)}(1-f_{j,t_i}^{(r)})\left( 1-\left( 1-\frac{1}{N_e^{(r)}}\right) ^{t_{i+1}-t_i}\right) \end{aligned}$$

with *k* being the total number of sequenced time points, $t_0=0$, $t_{k-1}=T$ being the first and last time points respectively as before. In practice, the effective population size $N_e$ will often be unknown and needs to be estimated for instance by using the method proposed by [20]. In the special case where no information at intermediate time points is available we have $k=2$, as only the start and end time points are sequenced. The test is carried out for all null hypotheses $H_{0j},$ $j\in \{1,2,\ldots ,N_{{\text {Hap}}}\}$ provided in (4). This leads to $N_{{\text {Hap}}}$ *p* values that are combined by a suitable multiple testing procedure.

Algorithm 1 provides pseudocode that summarizes our approach. Details of the proposed multiple testing approach can be found in "Multiple testing procedure" section below.

### Multiple testing procedure

We carry out one hypothesis test for each hypothesis pair in (4) and want to test the global null hypothesis (i.e. the null hypothesis for all $j\in \{1,2,\ldots ,N_{{\text {Hap}}}\}$ ). To control the type I error, we need a proper multiple hypothesis testing procedure. In principle, Bonferroni tests [21] or the recently proposed approach by [22] outlined in Additional file 1: Section S.1 might be used. However, we found these methods to be quite conservative in most situations. Therefore, we focus on more powerful approaches such as the omnibus test [23] and the harmonic mean *p* value [24]. Although no theory ensures type I error control for these methods under dependence, most of our simulated scenarios did not lead to violations. However, we observed type I error probabilities that slightly exceeded the significance threshold for both tests in the case of one replicate population, a small number of haplotypes, and known haplotype frequencies. See "Type I error control" section for details.

Algorithm 1: Haplotype based selection testing
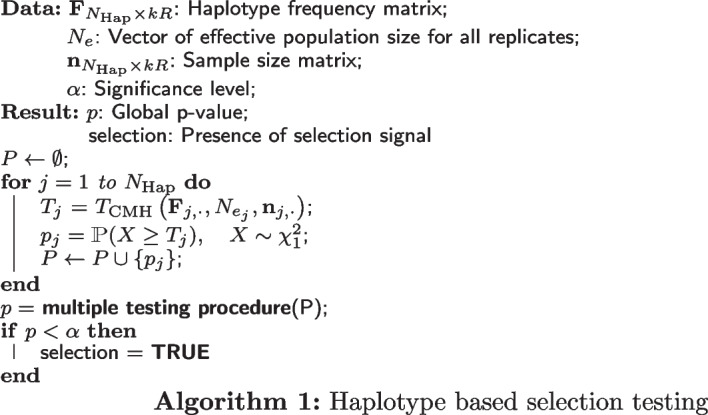


#### Omnibus test

The omnibus test proposed by [23] has originally been derived for independent *p* values, and was shown to provide good power under various deviations from the global null hypothesis. For sorted *p* values $\{p_{(j)}\}_{j\in \{1,2,\ldots ,N_{{\text {Hap}}} \}}$ such that:

7 $$\begin{aligned} p_{(1)}\le p_{(2)}\cdots \le p_{(N_{{\text {Hap}}})} \end{aligned}$$

the L-statistic $S_i$ is computed by adding up transformed *p* values up to rank *i*. Here we use the proposed default transformation (negative logarithm) leading to

8 $$\begin{aligned} S_i=\sum _{j\le i}-\log (p_{(j)}) \end{aligned}$$

with the test statistic *T* being:

9 $$\begin{aligned} T=\max _{i}G_i(S_i) \end{aligned}$$

and $G_i$ the cumulative distribution of $S_i$ under the global null hypothesis of neutrality. Note that the assumption of independent *p* values is not fulfilled in our context, as the haplotype frequencies at any given time point add up to 1. We observed violations in terms of type I error control only under a few scenarios, see "Influence of the model parameters" section  for details.

#### Harmonic mean *p* value (HMP)

Another combination method, the harmonic mean *p* value proposed in [24] is given by

10 $$\begin{aligned} \mathring{p} = \frac{N_{{\text {Hap}}}}{\sum _{j=1}^{N_{{\text {Hap}}}}1/p_j} \end{aligned}$$

for equal weights. The combined *p* value *p* is then calculated as

11 $$\begin{aligned} p=\int _{1/\mathring{p}}^\infty f_{\text {Landau}}\left( x|\log N_{{\text {Hap}}} +0.874, \frac{\pi }{2}\right) \,dx \end{aligned}$$

with $f_{\text {Landau}}$ being the density function of a Landau distribution.

While [25] showed that the type I error is not controlled under some dependence structures, we did not observe any large violations under our considered scenarios. See "Type I error control" section for further details.

### SNP based testing

Both haplotype and SNP based tests for selection usually rely on allele frequency changes. However, with SNPs only two alleles are usually considered in the literature whereas here multiple haplotypes can be present in the region of interest. The allele frequency changes of the two alleles are then tested in a similar fashion as we introduce here with the haplotype frequency changes. A review of commonly used methods for SNP based testing can be found in [4]. We compare our proposed haplotype based test with the SNP based approach presented in [3]. Time series data and replicate populations can be accounted for by this test as in our proposed approach and our haplotype based test is constructed following a similar rationale as the test proposed in [3]. When applying the SNP based test we only show results under the Benjamini & Hochberg multiple testing correction [26]. Indeed, this is a commonly used multiple testing approach in practical applications with allele frequency testing (see e.g.  [27, 28]). We also considered the multiple testing approaches presented in "Multiple testing procedure" section. For SNP based tests, the harmonic mean *p* values perform similarly to the Benjamini & Hochberg correction since they both aim to control the false discovery rate. On the contrary, the high dependence between SNP based *p* values causes consistently large violations of type I error when using the omnibus multiple testing correction. Thus we only show results under the Benjamini & Hochberg correction.

### Testing for the number of selected haplotypes

In association studies and genomic prediction, regression models are often used to explore the influence of haplotypes on a phenotype [7, 29]. While such a response variable is lacking in our setup, further information about the number of selected haplotypes is of interest in our context. Therefore we propose a follow up test for this purpose. As with forward selection methods in regression models, our approach proceeds in a stepwise fashion. At each step a test is carried out at level $\alpha ,$ and the procedure stops once no more rejection is necessary. We call a haplotype to be positively selected if there is at least one other haplotype with lower fitness.

A rejection of the global hypothesis (4) implies that there is at least one selected haplotype. To investigate whether there are further selected haplotypes, we identify the haplotype $m_1$ that provides the maximum change in frequency over time, normalised by variance and gives the largest contribution to the rejection of the hypothesis:

12 $$\begin{aligned} m_1:\max _{m_1}\frac{f_{m_1,T}-f_{m_1,0}}{\sqrt{\sigma _{drift,\Delta N_e}}}. \end{aligned}$$

We use

13 $$\begin{aligned} \sigma _{drift,\Delta N_e}:=\sum ^{k-2}_{v=0}f_{i,t_v,1}(1-f_{i,t_v,1})\left( 1-\left( 1-\frac{N_{e_v}^{-1}+N_{e_{v+1}}^{-1}}{2}\right) ^{t_{v+1}-t_v}\right) \end{aligned}$$

in situations when the true haplotype frequencies are known. This drift variance estimate is more complex than (6). However, it will simply reduce to $\sigma _{{\text {drift}}}$ in cases where $N_e$ is constant s.t. $N_{e_v}=N_{e_{v+1}}$, $\forall v$. For scenarios where haplotype frequencies are estimated, this variance term will need to change accordingly, see Additional file 1: Section S.2.5 for more details.

If $f_{{m_1},T}<1$, we test whether there are further selected haplotypes. For this purpose, we remove haplotype $m_1$ and test for fitness differences among the remaining haplotypes:

14 $$\begin{aligned} \begin{aligned} \mathbf {H_0}: \bigcap \nolimits _{j\in \{1,2,\ldots ,N_{{\text {Hap}}}\}\setminus \{m_1\}} \left\{ \phi _j=\bar{\phi }_{\{{1,2,\ldots ,N_{{\text {Hap}}}\}\setminus \{m_1\}}}\right\} \\ \mathbf {H_1}: \bigcup \nolimits _{j\in \{1,2,\ldots ,N_{{\text {Hap}}}\}\setminus \{m_1\}} \left\{ \phi _j\ne \bar{\phi }_{\{{1,2,\ldots ,N_{{\text {Hap}}}\}\setminus \{m_1\}}}\right\} \\ \end{aligned} \end{aligned}$$

where $\bar{\phi }_{\{{1,2,\ldots ,N_{{\text {Hap}}}\}{\setminus } \{m_1\}}}$ is the mean fitness of all haplotypes except $m_1$. We furthermore renormalise the remaining haplotype frequencies to add up to 1 at any time point:

15 $$\begin{aligned} r_{i,t_v,1}= f_{i,t_v}c^{(m_1)}_{t_v} \end{aligned}$$

where $c^{(m_1)}_{t_v}=[\sum _{j\in \{1,2,\ldots ,N_{{\text {Hap}}}\}\setminus \{m_1\}}f_{j,t_v}]^{-1}$. We also recompute $N_e$ separately for each time point to take the removal of haplotype $m_1$ into account:

16 $$\begin{aligned} N_{e_v}^{(m_1)}=\sum _{j\in \{1,2,\ldots ,N_{{\text {Hap}}}\}\setminus \{m_1\}}f_{j,t_v}N_e \end{aligned}$$

We then test for selection, but replace the variance caused by drift, $\sigma _{{\text {drift}}}$ in $T_{{\text {CMH}}}$ by $\sigma _{drift,\Delta N_e}$. If the null hypothesis is rejected, we claim that there are at least two selected haplotypes in the population.

The above method is then iterated to test for further selected haplotypes. For this purpose, we find $m_2, m_3,\ldots$ respectively by ranking the normalised differences in frequency change over time and testing the hypothesis:

17 $$\begin{aligned} \begin{aligned} \mathbf {H_0}: \bigcap \nolimits _{j\in \{1,2,\ldots ,N_{{\text {Hap}}}\}\setminus \{m_1,m_2,\ldots \}} \phi _j=\bar{\phi }_{\{1,2,\ldots ,N_{{\text {Hap}}}\}\setminus \{m_1,m_2,\ldots \}}\\ \mathbf {H_1}: \bigcup \nolimits _{j\in \{1,2,\ldots ,N_{{\text {Hap}}}\}\setminus \{m_1,m_2,\ldots \}} \phi _j\ne \bar{\phi }_{\{1,2,\ldots ,N_{{\text {Hap}}}\}\setminus \{m_1,m_2,\ldots \}} \end{aligned} \end{aligned}$$

as long as the previous null hypothesis is rejected. The method of normalisation, the $N_e$ computation, and the testing procedure are analogous to before.

If replicates are present, the largest change in haplotype frequency might not be consistent across all replicates. We therefore propose the following criterion to remove haplotypes:

18 $$\begin{aligned} m_1:\max _{m_1}\sum _{r=1}^R \frac{f^{(r)}_{m_1,T}-f^{(r)}_{m_1,0}}{\sqrt{\sigma _{drift,\Delta N_e}^{(r)}}} \end{aligned}$$

Further haplotypes are excluded in an analogous way. The values of $N_{e_v}^{(m_1)}$ and the frequency normalisation will then be calculated separately for each replicate.

### Pairwise test for different fitness across haplotypes

As further post hoc tests, we consider pairwise comparisons for differences in the fitness between haplotypes *i* and *j*:

19 $$\begin{aligned} \begin{aligned} \textbf{H}_{0_{i,j}}: \phi _i=\phi _j\\ \textbf{H}_{1_{i,j}}: \phi _i\ne \phi _j \end{aligned} \end{aligned}$$

We test this hypothesis pair, if their frequencies satisfy $\sum _{r=1}^R\left( f^{(r)}_{i,T}+f^{(r)}_{j,T}\right) \ne 0$. Given the haplotype frequency matrix $\textbf{F}$, for some pair of haplotypes *i* and *j* at replicate *r*, we normalise their frequencies to add up to one. For $l\in \{i,j\}$, we set $f^{(r)^{{\text {norm}}}}_{l,t_v}= f^{(r)}_{l,t_v}c_t^{(r)^{{\text {norm}}}}$, where $c^{(r)^{{\text {norm}}}}_{t_v}=(f^{(r)}_{i,t_v}+f^{(r)}_{j,t_v})^{-1}$ is the normalising constant of replicate *r* at generation $t_v$. Furthermore $N_{e_v}^{(r)^{{\text {norm}}}}$ at time point $t_v$ is computed as

20 $$\begin{aligned} N_{e_v}^{(r)^{{\text {norm}}}}=(f^{(r)}_{i,t_v}+f^{(r)}_{j,t_v})N_e^{(r)}. \end{aligned}$$

Since this will usually cause a changing $N_e$, we replace the drift variance by $\sigma _{drift,\Delta N_e}$ when applying our proposed test (5) to the haplotype pair. To control the false discovery rate, a Benjamini & Hochberg multiple testing correction will also be applied to the *p* values obtained from all considered pairs.

### Testing when many haplotypes are present

Scenarios with many haplotypes tend to lead to low power when using haplotype based testing. This is due to a large number of individual tests and the small haplotype frequencies. One way to resolve this issue is through the combination of haplotype frequencies. Intuitively, this can be achieved through the removal of SNPs, such that several haplotypes will become identical. Here, we propose two haplotype combination methods to improve the performance of our haplotype based tests. Several other approaches for haplotype reduction have been used in other fields such as GWAS, one example is haplotype clustering as in [30]. Since our focus here is on haplotype based testing, we do not provide an extensive comparison of available methods. However, we found the two procedures outlined below to work well in our context.

First, we propose an intuitive approach that combines haplotypes using individual SNP based tests. We use the *p* values of these tests without multiple testing corrections and retain SNPs with *p* values below some given threshold $\beta$. The haplotypes that become identical after SNP removal are then combined. A similar approach has been proposed in [31] to identify a selected haplotype. A detailed explanation of this approach is provided in Additional file 1: Section S.3.

Another possible approach relies on haplotype blocks obtained via techniques commonly used in GWAS [10]. A haplotype block may be defined as a contiguous region of SNPs that are in high linkage disequilibrium with each other with little evidence of recombination within the region [32]. We use a normalised version of the coefficient of linkage disequilibrium proposed by [33] and follow the approach by [32] to determine haplotype blocks. Our proposed haplotype based selection test is then applied to each of the combined haplotype block frequency matrices. An extra layer of between blocks multiple testing corrections is then needed. We refer to Additional file 1: Section S.4 for more details.

## Results

### Simulation experiments

In this section we present the results of an extensive simulation study where we analyse the performance of our proposed tests from "Methods" section under different scenarios. First, "Proof of concept" section provides a proof of concept, illustrating some potential advantages of our proposed haplotype based test compared to SNP based testing for selection in a typical experimental evolution scenario. Then we consider how the choices of the experimental design ("Influence of the model parameters" section) and of the model organism ("Diploid populations" section) can affect the power of our test compared to a SNP based test. Lastly, "Testing for the number of selected haplotypes, Pairwise test, and Experimental designs involving many haplotypes" sections illustrate the results of the extensions of our proposed test discussed in "Testing for the number of selected haplotypes, Pairwise test for different fitness across haplotypes, and Testing when many haplotypes are present" sections  respectively, and "Type I error control" section discusses type I error control of our proposed methods.

#### Data and simulation setup

Our simulation studies are inspired by the experimental setups described in [34] and in [16]. For our simulations related to the first setup, we randomly selected $N_{{\text {Hap}}}$ different haplotypes (from the iso-female lines) and $N_{SNP}$ SNPs (from a locus consisting of 500 SNPs). Starting from the chosen founder haplotypes, we simulated evolve and resequence experiments with and without selection by generating multinomial haplotype frequency changes along generations using Eq. (2).

All frequencies are assumed to be known unless otherwise stated. For haplotype frequencies, we set the starting frequencies at time point 0 to be equal, such that each haplotype has a frequency of $\frac{1}{N_{{\text {Hap}}}}$. As discussed in Additional file 1: Section S.13, and in "Real data application" section, however, our methods may also be used with arbitrary, unequal starting frequencies.

Under selection, we consider scenarios involving both one and more than one positively selected SNP. With $n_{sel}\ge 1$ selected SNPs, we randomly choose positions $J=(j_1,j_2,\ldots ,j_{n_{sel}})^\intercal$ for the selected SNPs. The corresponding vector $S=(s_1,s_2,\ldots ,s_{n_{sel}})^\intercal$ denotes the selection coefficients of these SNPs. Assuming additive fitness effects, this leads to a fitness vector $\Phi$ (see Eq. (3)) with components

21 $$\begin{aligned} \phi _i = 1+\sum _{j\in J \text { s.t. } h_{j,i}=1} s_j \end{aligned}$$

for haplotype *i* ($$1\le i\le N_{{\text {Hap}}}$$).

We then simulate haplotype frequencies up to $(k-1)\times \Delta t$ generations. Given a haplotype structure matrix $\textbf{H}$ and a haplotype frequency matrix $\textbf{F}^{(r)}$ for replicate *r*, the allele frequency matrix $\textbf{A}^{(r)}$ for this replicate can be calculated as:

22 $$\begin{aligned} \textbf{A}^{(r)} = \textbf{HF}^{(r)} \end{aligned}$$

where each element $a_{i,j}$ denotes the allele frequency for SNP *i* at generation $t_j$. Furthermore, $\textbf{H} \in \{0,1\}^{I \times N_{Hap}}$ is constructed such that $\textbf{H}_{in} = 1,$ if haplotype *n* assumes the reference allele at SNP *i* and $\textbf{H}_{in} = 0$ otherwise. The columns of $\textbf{H}$ denote the haplotypes and the rows the SNPs. For scenarios where frequencies are estimated with a sample size of $n_{i,j}^{(r)}$, we construct the noisy haplotype frequency matrix $\hat{\mathbf{F}}^{(r)}$ by drawing its columns independently via multinomial sampling. The observed allele frequencies are then obtained as

23 $$\begin{aligned} \hat{\textbf{A}}^{(r)}= \mathbf{H} \hat{\mathbf{F}}^{(r)} \end{aligned}$$

Under pool sequencing with sequencing coverage $u^{(r)}_{i,j}$ for SNP *i* at time point $t_j$ and replicate *r*, we construct the noisy allele frequency matrix $\tilde{\textbf{A}}^{(r)}$ by drawing each element via binomial sampling using the respective sequencing coverage. With SNP based testing, these binomial variances are added as a component of variance to the denominator of the modified CMH tests (see Additional file 1: Section S.2.3). For the haplotype based test, we assume that the haplotype frequencies are estimated from the noisy allele frequencies $\tilde{\textbf{A}}^{(r)}$ by solving the regression model [35]. Other methods of haplotype frequencies may also be used, see for instance [36] that proposes an EM algorithm, or [37] for a maximum likelihood based approach.

24 $$\begin{aligned} \tilde{\textbf{A}}^{(r)}= \mathbf {H} \hat{\mathbf{F}}_P^{(r)}. \end{aligned}$$

The variances of the estimated regression coefficients $\hat{\mathbf{F}}_P^{(r)}$ as well as of their sums are then used as additional components of variance.

#### Proof of concept

As an initial illustration of our hypothesis test we consider 10,000 simulations from a window of 500 SNPs, mimicking an experiment with 10 replicates and a haploid population of size 1000, with a coverage of 50. Ten founder haplotypes are present in each population. We consider a scenario with 60 generations and with sequencing taking place every 10 generations. To simulate selection, one SNP is assumed to be beneficial. In our first example the SNP is private to one of the haplotypes and has a selective strength $s=0.02$. In the second example, the selected SNP is shared among five haplotypes and has a selective strength $s=0.03$. We chose these two selection regimes for illustration purposes, but similar conclusions can also be drawn when changing the selection strength.

**Fig. 1:**
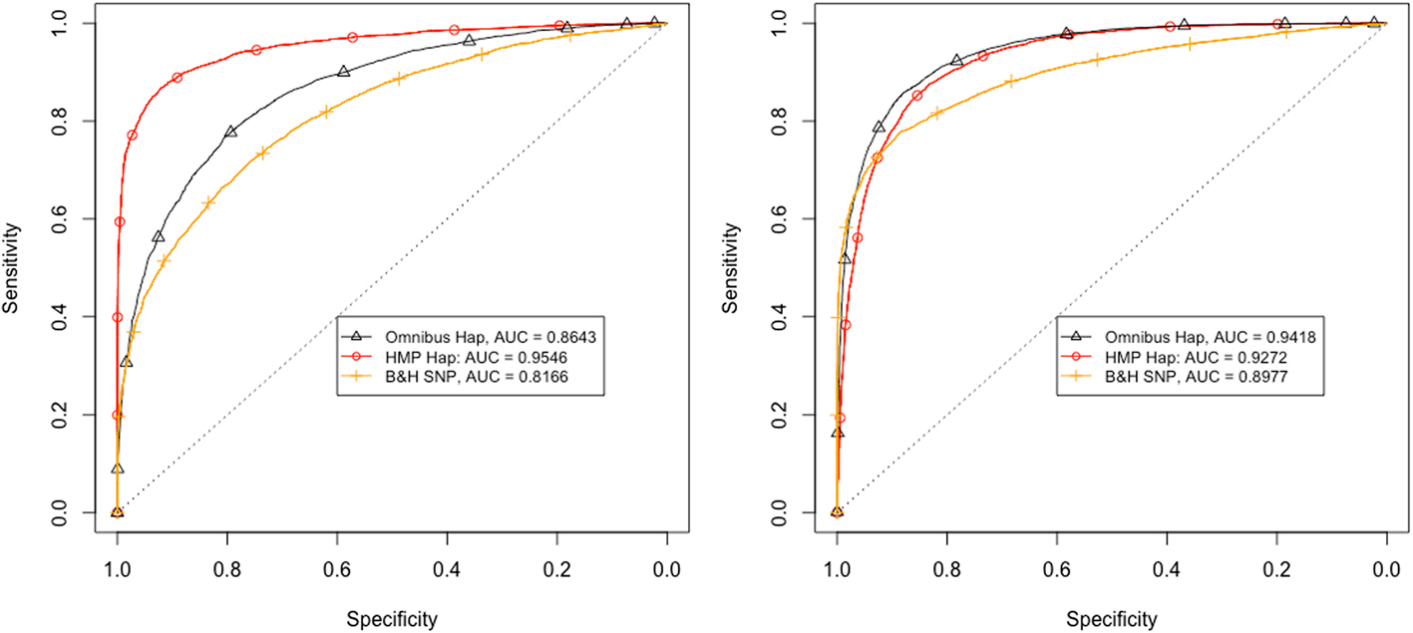
Results for two simulated examples that mimic a typical experiment. The receiver operating characteristic (ROC) curves of the haplotype and SNP based tests are shown for two scenarios: 10 replicate populations with 10 founder haplotypes each are simulated with a coverage of 50. In the left panel, one haplotype is beneficial with $s=0.02$. In the right panel $s=0.03$, and 5 haplotypes have a common selective advantage compared to the remaining populations

The receiver operating characteristic (ROC) curves for our proposed haplotype based test and SNP based test under the two scenarios are plotted in Fig. 1. We include results under the HMP and the omnibus *p* value combination methods.

The haplotype based tests have a higher area under the curve (AUC) than the SNP based test in both examples. The difference is particularly large under the scenario with one selected haplotype (left panel of Fig. 1). This demonstrates that our proposed approach is able to provide a considerable increase in power under some scenarios.

The harmonic mean *p* value combination method has particularly high power in scenarios with a single true alternative. Such a situation occurs both when one, or all but one haplotype are selected. On the other hand, the omnibus test performs better than HMP at intermediate numbers of selected haplotypes (see right panel of Fig. 1).

With an intermediate number of selected haplotypes, the advantage compared to SNP based testing is also smaller than for both a large and a small number of selected haplotypes. See Additional file 1: Figure S2 for results under scenarios without pool sequencing noise.

#### Influence of the model parameters

In experimental evolution, different organisms and experimental setups are chosen according to the aims of the experiment and the available resources [38]. Therefore, we discuss the impact of the experimental design on the performance of our haplotype based tests. We investigated the influence of each parameter described in "Data and Simulation setup" section on the performance of our proposed tests by considering a set of alternative values for each of them. The other parameters have been kept constant as listed in the reference Table 2. The results in this section assume known haplotype frequencies unless otherwise stated and are based on the test statistic provided in "Conclusion" section.

**Table 2:** Default parameter values for "Influence of the model parameters, Diploid populations, Testing for the number of selected haplotypes, Pairwise test, Experimental designs involving many haplotypes" sections and Additional file 1: Section S.6. Unless otherwise mentioned, Figures within these sections use parameters from this table when simulating results

Parameters		
$N_{SNP}$	500	Number of SNPs per haplotype
$N_{{\text {Hap}}}$	10	Number of haplotypes
*k*	7	Total number of sequenced time points
$\Delta t$	10	Number of generations between adjacent time points
$N_e$	1000	Effective population size
$h_{{\text {Sel}}}$	1	Number of selected haplotypes
*s*	0.03	Selective strength
*R*	3	Number of replicates
$\alpha$	0.05	Significance level
$n_{{\text {sim}}}$	10,000	Number of simulations per parameter set

**Fig. 2:**
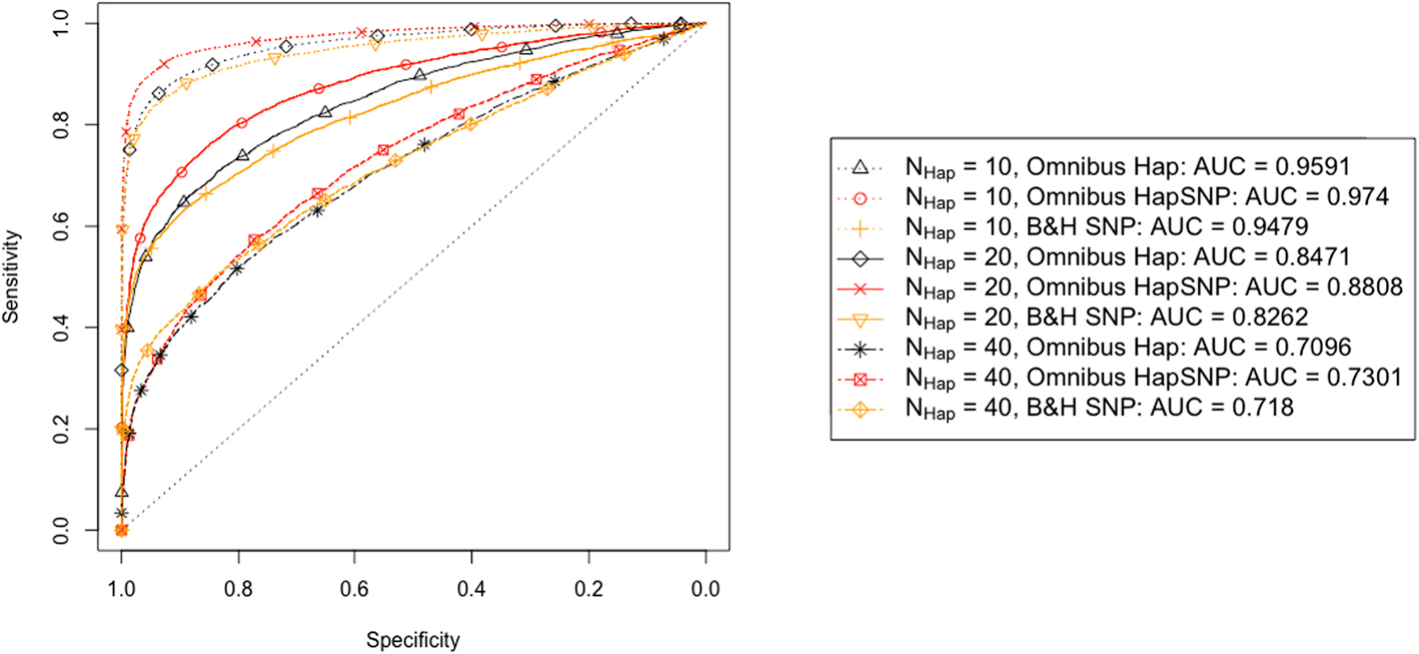
Results for 3 scenarios with different numbers of haplotypes. ROC curves of both haplotype and SNP based tests are shown for 10, 20 and 40 total haplotypes, with all other parameters default as outlined in Table 2

Figure 2 shows that the power of all tests decreases with an increasing number of initial haplotypes. We observe that the differences in AUC between haplotype and SNP based tests decrease as the number of haplotypes increases. When using the omnibus multiple testing correction, the SNP based approach also slightly outperforms our haplotype based test, if the number of starting haplotypes is large. This can be explained by the need of more multiple testing corrections due to the increase in the number of haplotypes.

As detailed in "Testing when many haplotypes are present" section, in scenarios with a large number of haplotypes the starting haplotype frequency is very small and thus the probability that the selected haplotype is lost due to drift in the early phase of the experiment is high. On the other hand, when a large number of haplotypes have high fitness and their fitness values are identical, the chance that the neutral haplotypes are lost at the beginning of the experiment is high. This leads to a scenario where all haplotypes have the same selective strength, and thus there is no selective advantage for any haplotype, resulting in a low power of all considered tests.

We show in Additional file 1: Figure S2, that despite a small initial increase, more selected haplotypes will result in lower power for all tests. For the haplotype based tests, the two multiple testing corrections outperform each other in different situations. While HMP performs best with very few and with many selected haplotypes, the omnibus test works best with an intermediate number of selected haplotypes.

Additional file 1: Section S.6 provides simulation results for different values of the number of replicates, the selective strength, the number of generations, and the effective population size. As previously observed with SNP based tests [39], these results confirm that more replicate populations, a higher selective strength, more generations, and a higher effective population size lead to an increase in power for all tests. The presence of more replicates especially, benefits haplotype based test much more in terms of power compared to the SNP based test. We also observed that the haplotype based tests perform better than the SNP based approach under all considered scenarios.

For a scenario with unequal starting haplotype frequencies and either three or four founder haplotypes, see Additional file 1: Section S.13. All the other parameters are as in Table 2, except with no replicate population. We note that also in this situation the haplotype based test retains good power compared to the SNP based test in most scenarios. There were some scenarios, with little difference in power however. In such cases, our simulations did not lead to statistically significant differences in AUC at level $\alpha =0.05$. Overall the starting frequencies of the haplotypes influence the relative performance of haplotype based tests. If there is one selected haplotype, the haplotype based test performs best either when the starting frequency of the selected haplotype is large or small. For two and three selected haplotypes, it seems to perform best when the combined starting frequency of selected haplotypes is large.

We finally studied the effect of the number of SNPs in the considered window. As shown in Additional file 1: Section S.6, the haplotype based test is invariant with respect to this parameter. On the other hand, the power of the SNP based test decreases with an increasing number of SNPs. This is due to the more stringent multiple testing correction needed. In principle, the advantage of haplotype based testing increases with the window size. However, with large windows recombination becomes increasingly relevant for haplotype based testing. Thus, if the window size is too large then haplotype based testing becomes unfeasible due to a large number of haplotypes.

In experimental evolution, haplotype frequencies are often unknown. If estimates are used instead, and their errors are non-negligible, we propose to use the test statistics introduced in Additional file 1: Sections S.2.1 and S.2.2, and S.2.3 instead. They account for the additional variance incurred by haplotype frequency estimates. Both SNP and haplotype based testing will need to take the additional variance into account, if the exact allele frequencies are unknown and replaced by estimates. Notice however, that pool-sequencing noise can be reduced with the haplotype based approach as it combines information across SNPs, for instance via regression. In Additional file 1: Figure S13, we consider cases where all frequencies are estimated with a sample size of 500, and with various sequencing coverage values between 50 and 450. With data obtained via pool sequencing, the power decreases only for SNP based testing. Especially for low sequencing coverage, haplotype based testing therefore provides a considerable advantage.

Since both drift and sampling variance affect all tests in similar ways, sampling variation will decrease the power overall. Additional file 1: Section S.6 provides an illustrative example.

#### Diploid populations

**Fig. 3:**
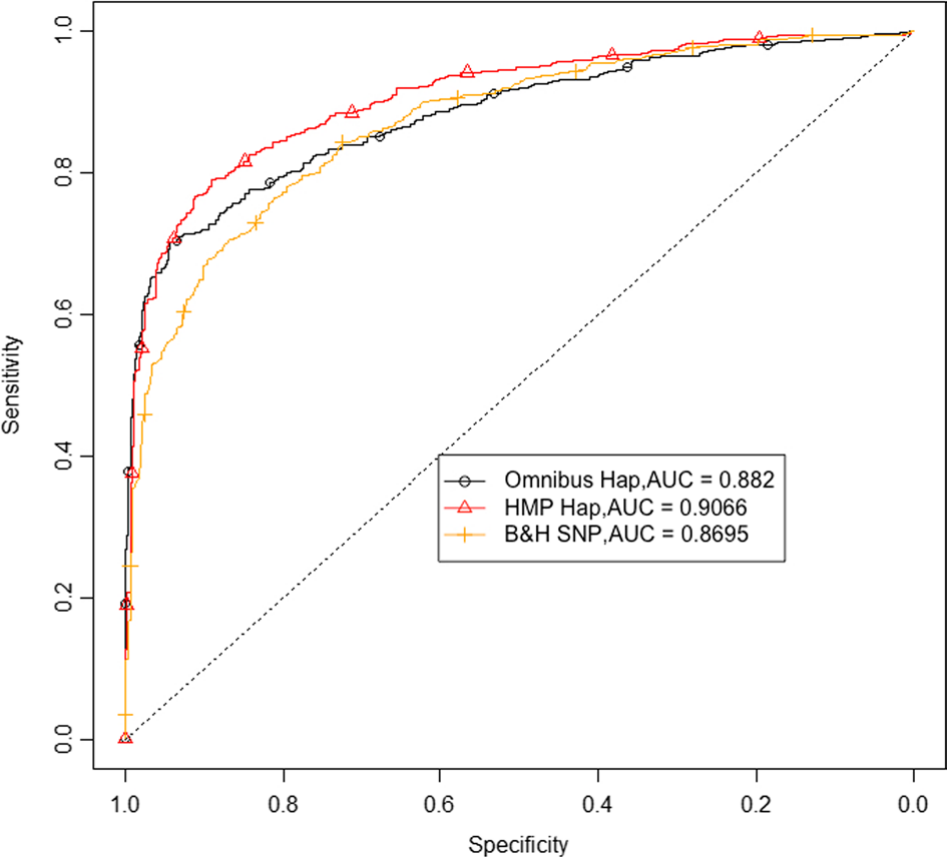
Results for a diploid population. ROC curves of both haplotype and SNP based tests are shown for a diploid population. Here, the number of simulations is reduced to 1000, with all other parameters outlined in Table 2

Here, we evaluate the performance of our proposed methods on a diploid population instead of a haploid one. The population is simulated using the software [40] with 1000 simulated samples, and other parameters outlined in Table 2. Additive genetic effects are assumed. The results for the diploid population (Fig. 3) are similar to the haploid case (Fig. 2), where the haplotype based tests outperform the SNP based test in terms of AUC. The haplotype based test using HMP as *p* value combination method performs best in line with previously seen haploid results with one selected haplotype. Since heterozygous individuals with one selected allele have a selective advantage of only *s*/2 when assuming additive effects, the power of all tests is lower compared to the haploid case.

#### Testing when the effective population size is unknown

In real data, the effective population size will be typically unknown. Thus the true $N_e$ needs to be replaced by an estimate such as the one proposed in [20]. Since such methods are generally derived based on the absence of selection, the estimates will be biased, if they rely on a substantial number of selected SNPs. This bias will lead to an underestimated $N_e$, and will make our approach more conservative.

**Fig. 4:**
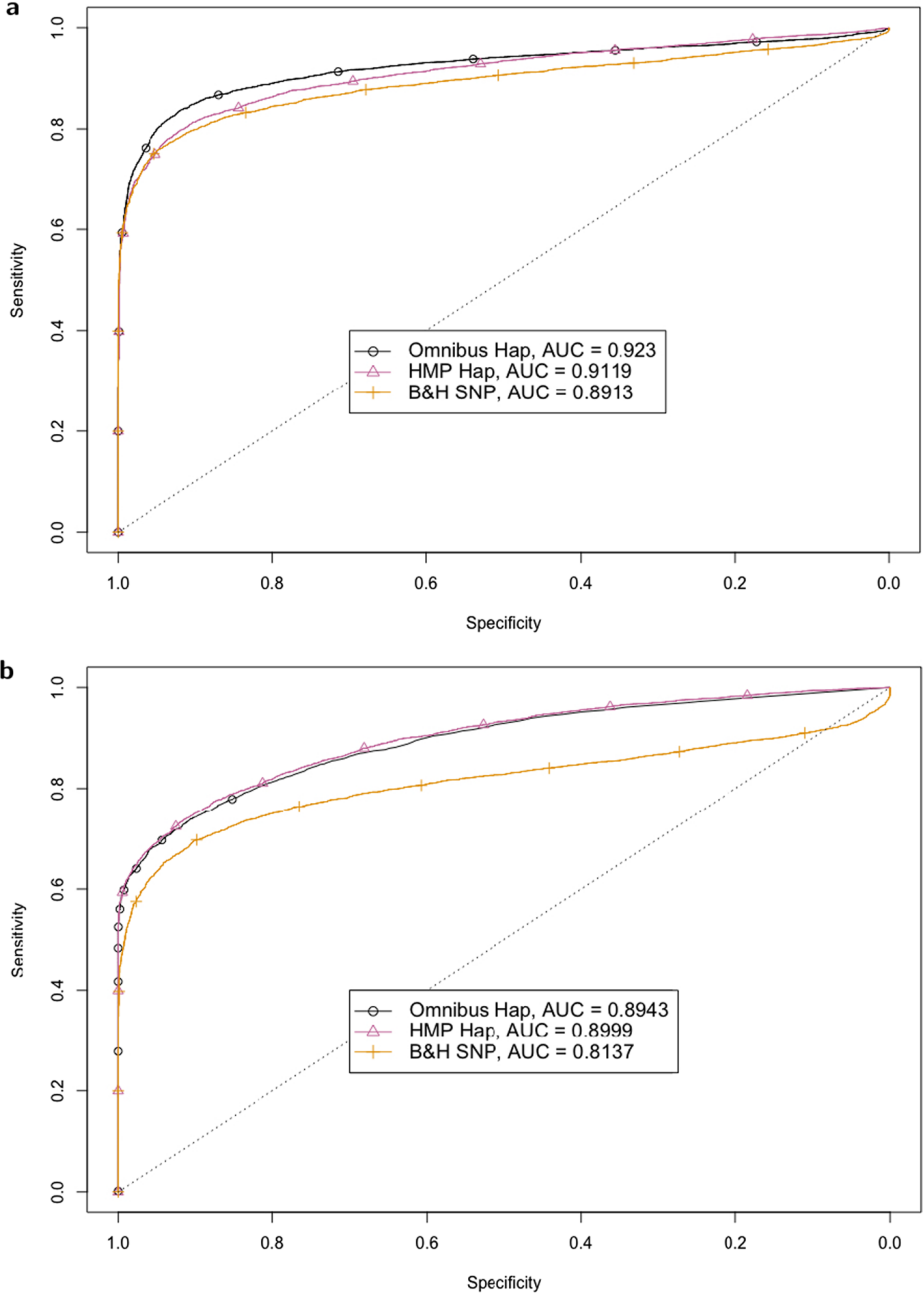
Results for estimated effective population size. ROC curves of both haplotype and SNP based tests are shown for a scenario where $N_e$ is estimated. Here, the ROC curve is obtained using the same parameters as in the right panel of Fig. 1, but with $N_e$ estimated. In the top figure, $N_e$ is estimated from SNPs taken from an independent neutrally evolving window. In the bottom figure, $N_e$ is estimated using the testing window regardless of the presence of selection

The amount of bias will depend on the proportion of non-neutral SNPs used to estimate $N_e$ and their selective strength. Here we consider 2 extreme scenarios, one where $N_e$ is estimated from SNPs taken from an independent neutrally evolving window, and another where $N_e$ is estimated from the tested window. Thus, if the tested window is affected by selection, the $N_e$ estimate will be systematically too small. This can be seen in Fig. 4b, where the AUC is considerably smaller compared to Fig. 4a where $N_e$ is estimated from neutral data. Notice however, that the loss in power is much larger for the SNP based test than for the haplotype based tests. Compared to the case where $N_e$ is known (Fig. 1), estimating $N_e$ leads to a lower AUC pointing towards a lower classification accuracy. Naturally, this decreased accuracy will depend on the variance of the $N_e$ estimate, which will be smaller when larger genomic regions are used in the estimation process.

#### Testing for the number of selected haplotypes

The knowledge of the number of selected haplotypes when selection is present is of interest in practical applications when researchers try to better understand the genomic architecture of adaptation in experimental evolution. To investigate the practical performance of the test proposed in "Testing for the number of selected haplotypes" section, we simulated a scenario with 5 founder haplotypes, some of them selected with $s=0.05$, that otherwise follows the population parameters in Table 2. We generate 10,000 simulation runs for each considered number of selected haplotypes. If selection is detected by our haplotype based test with the omnibus *p* value combination method, we apply our proposed iterative test from "Testing for the number of selected haplotypes" section.

**Fig. 5:**
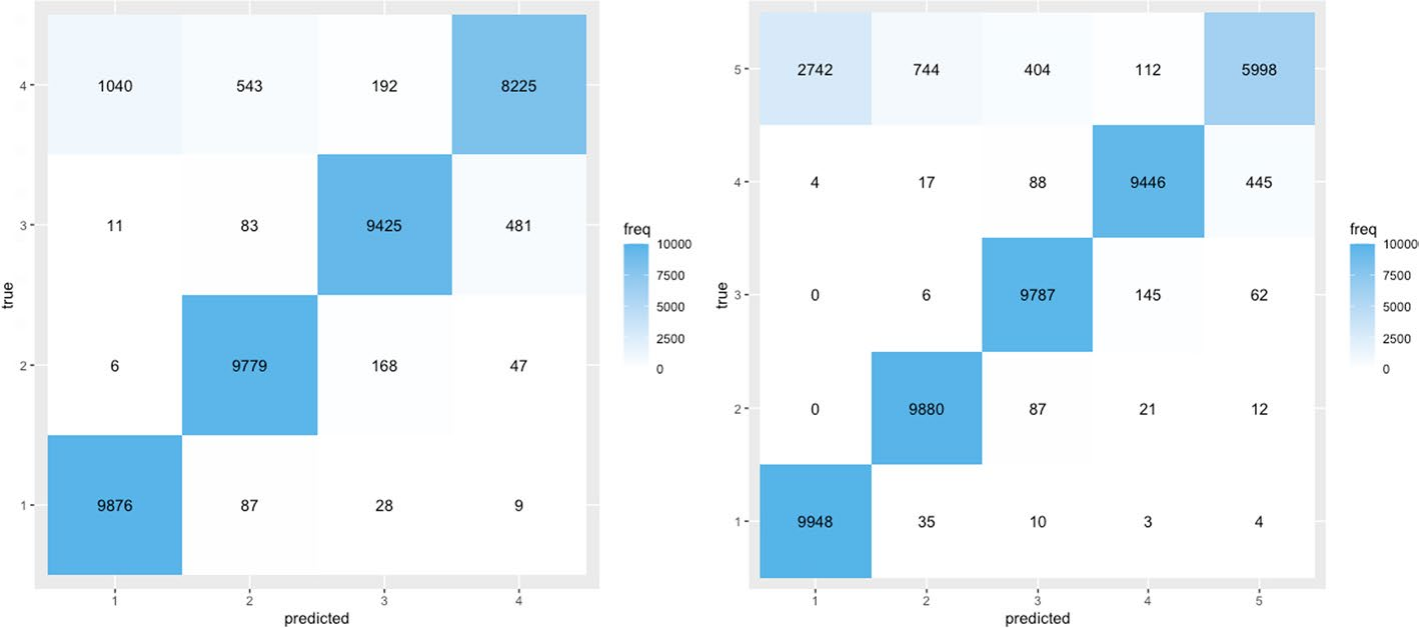
Results showing the prediction accuracy of haplotype based iterative testing. The omnibus method is used for multiple testing corrections in all scenarios. The heat map has been obtained using 5 founder haplotypes and a selection strength of $s = 0.05$, with all other parameters default as outlined in Table 2. In the left panel, the haplotype with the strongest signal is always removed as selected. In the right panel, the test is unconditional

Figure 5 illustrates that this test is able to accurately predict the number of selected haplotypes for scenarios with different true numbers of selected haplotypes given that selection is strong enough. The left panel presents results conditional on the presence of selection, and the right panel unconditionally.

The scenario with one selected haplotype, for instance, is identified correctly in more than $98\%$ of cases both conditionally and unconditionally. We show similar results using the HMP combination method in Additional file 1: Figure S15.

#### Pairwise test

Under the simulation scenarios detailed in "Testing for the number of selected haplotypes" section, we also explored the performance of the pairwise post hoc test statistic for differences in fitness proposed in "Pairwise test for different fitness across haplotypes" section.

**Fig. 6:**
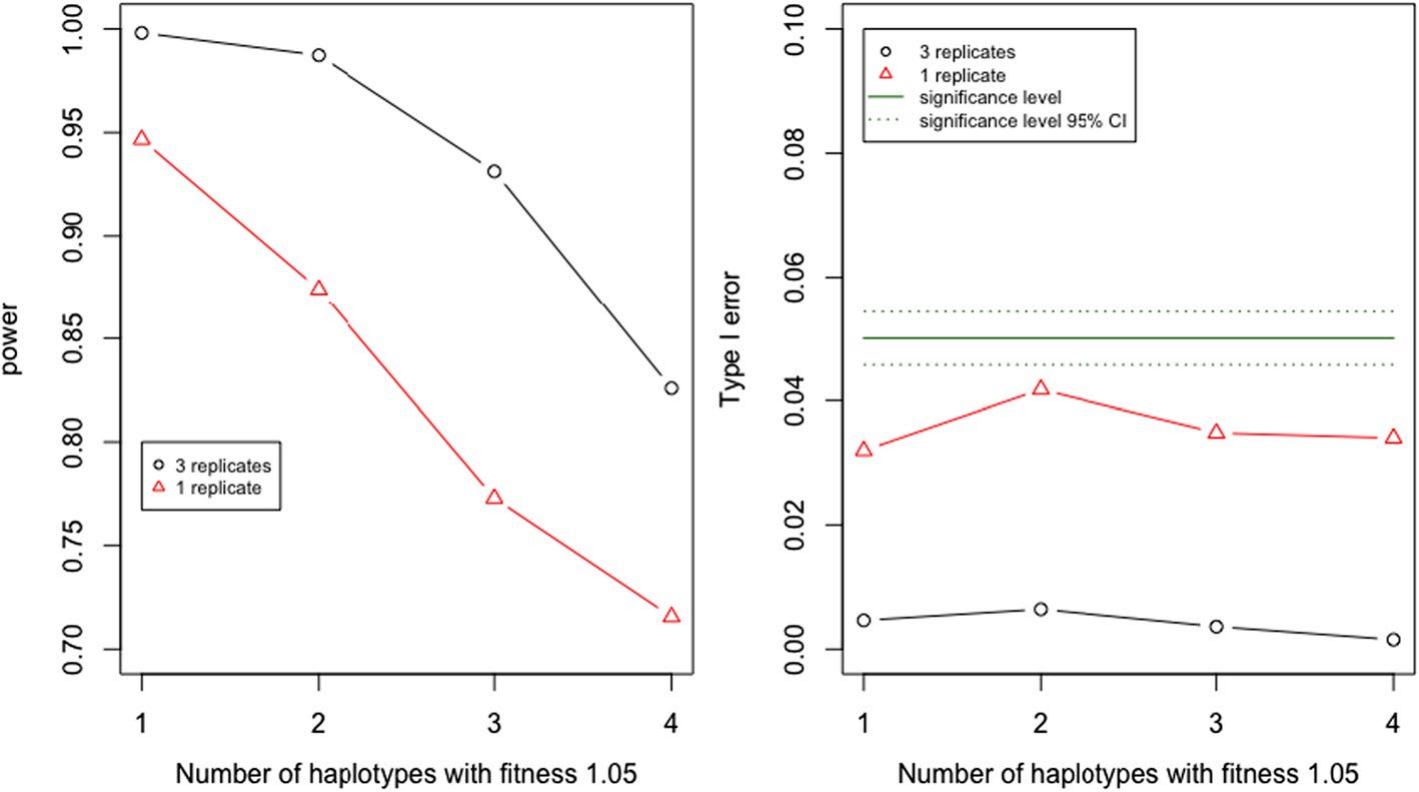
Pairwise tests for differences in fitness for different numbers of selected haplotypes. The displayed probabilities are conditional on the rejection of the initial test for selection. Results based on simulated data using 5 founder haplotypes, selection strength $s=0.05$, different numbers of selected haplotypes, and 1 or 3 replicate populations, with all other parameters default as outlined in Table 2. Type I errors occur when the test between two haplotypes of equal fitness rejects the null hypothesis. B &H is used for multiple testing corrections

Figure 6 provides results on the power and the type I error probability, conditional on the rejection of the initial test. Both under the scenarios involving one and three replicate populations, the power of predicting fitness differences between haplotype pairs is high and the type I error probability is controlled. Again, the power is higher with more replicate populations. We observe a decrease in power when the number of selected haplotypes increases.

We also investigate the behaviour of the pairwise test under a scenario where all haplotypes differ in fitness. We assign fitness values of 1, 1.02, 1.04, 1.06, and 1.08 respectively to the five considered haplotypes. As one might expect, Additional file 1: Figure S18 indicates that power increases with an increasing difference in fitness between the tested haplotypes. For more details on this parameter set, see Additional file 1: Section S.10 and S.11.

#### Experimental designs involving many haplotypes

As discussed already in "Influence of the model parameters" section, the power of our approach decreases with an increasing number of haplotypes. Here we investigate whether the methods proposed in "Testing when many haplotypes are present" section to reduce the number of haplotypes helps to resolve this problem.

**Fig. 7:**
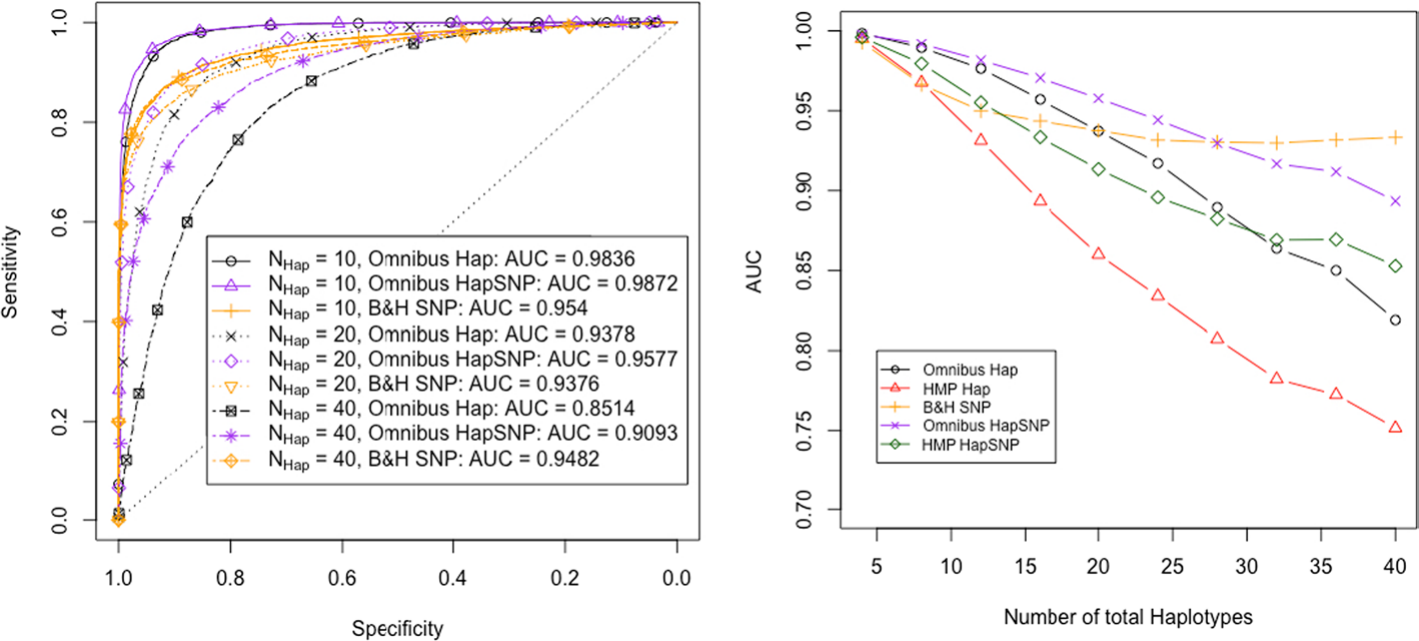
Results for different total numbers of haplotypes. In the left panel, ROC curves are plotted for the original haplotype, HapSNP, and SNP based test under various choices of $N_{{\text {Hap}}}$. HapSNP refers to the method of using the SNP based test to reduce the haplotype number prior to haplotype based testing outlined in "Testing when many haplotypes are present" section. Here, results are shown for 10, 20, and 40 starting haplotypes. The number of selected haplotypes is set to $h_{{\text {Sel}}} = N_{{\text {Hap}}}/2$. All other parameter values can be found in Table 2. In the right panel, the AUC of the ROC curves is plotted independently of the starting haplotype number. The other parameter values are identical to those used in the left panel

To better understand the interplay between the number of founder haplotypes and that of selected haplotypes, we plot results with an intermediate number of selected haplotypes for different numbers of founders in the left panel of Fig. 7. There, $h_{{\text {Sel}}}=N_{{\text {Hap}}}/2$, while the rest of the parameters are as in Fig. 2. As suggested by the results in Fig. 7, the haplotype based tests lose their advantage compared to SNP based tests already at 20 founder haplotypes instead of around 40 in Fig. 2. Indeed, if many haplotypes have a similar selective advantage, the signal that can be captured by the haplotype based test is diluted. This emphasises that haplotypes should be combined as explained in "Testing when many haplotypes are present" section.

We see that the SNP based combination method (HapSNP) is still able to retain an AUC advantage at 20 haplotypes. Both panels of Fig. 7 also show that the SNP based combination method improves the power of the considered haplotype based test consistently. The advantage of haplotype based testing is retained this way also for designs involving a considerably larger number of haplotypes compared to tests that do not operate on a reduced set of haplotypes. The SNP based combination method first reduces the number of haplotypes by removing SNPs, and then applies our proposed haplotype based test. The type I error plot in Additional file 1: Section S.12 suggests that the type I error probabilities are also controlled with this approach.

This approach only requires a minimal increase in terms of computational cost and performs well when the SNP based test is able to identify a reasonable number of individually significant SNPs. When this is not the case, we propose a more computationally demanding modification. This approach reduces the number of haplotypes by creating haplotype blocks and then performing the test for selection on the haplotype blocks. Additional file 1: Section S.12 provides results for a scenario with 100 haplotypes, 50 selected haplotypes, and only one population. Here, the haplotype based test combined with the haplotype block based approach performs equally well or better than SNP based tests despite the large haplotype number. This is due to the haplotype block based test being mostly invariant to changes in the number of haplotypes (see Additional file 1: Section S.12).

Additional file 1: Figure S25 provides an example where 30 haplotypes are present, one of them selected. We notice that the SNP based combination method does not perform as well here, in terms of power in subsequent testing. To investigate this further, we considered several significance thresholds which lead to different sets of considered SNPs. However, Additional file 1: Figure S26 shows that the choice of threshold seems to have little impact on the performance.

#### Type I error control

As can be seen in Additional file 1: Section S.6, haplotype based tests provide well controlled type I error probabilities, in most of the scenarios we explored. While most changes in parameter value have little to no effect on the type I error of the test, the number of replicates, the number of haplotypes, and whether the frequencies are known or estimated can have a noticeable impact.

The largest violation in terms of type I error we encountered during our extensive simulations was with the combination of 1 population, 3 to 4 haplotypes, and known haplotype frequencies (Additional file 1: Figure S1). Even for these scenarios, the type I error probabilities stay below 0.07, given a significance threshold of 0.05 for the *p* values.

We also notice that the CMH test is more conservative than the chi-squared test, causing a drastic decrease in type I error probability when replicates are present. This conservativeness is explored in further detail in Additional file 1: Section S.11.

The post hoc tests share similar characteristics in terms of type I error probabilities. The pairwise test tends to have a lower type I error probability compared to the initial test preceding the post hoc procedure (Fig. 6), whilst the test for the number of selected haplotypes tends to have a somewhat higher type I error probabilities than the initial test (Fig. 5). Indeed, for the pairwise tests we did not observe any violations of the level $\alpha$ even under the most liberal scenario. On the other hand, Additional file 1: Figure S14 suggests that the true number of haplotypes is over-estimated in about 11% of cases at worst.

One scenario when such a claim does not necessarily hold is due to the type I error being computed conditional on the rejection of the null hypothesis of the haplotype based test. These conditional type I errors often have higher probabilities compared to unconditional type I errors, which are not dependent on the rejection of the null hypothesis. This is the case in particular under weak selection, where a larger proportion of the initial rejections is caused by inflated effect sizes due to random errors. Due to such filtering, the conditional type I error can exceed the desired threshold, while the unconditional type I error is well under control. With one selected haplotype, Additional file 1: Figure S17 provides an example that shows that the conditional type I error is not under control under a very low selective strength $s=0.01$.

With the haplotype block based tests there are two levels of multiple testing correction, one within and one between the haplotype blocks. We suggest the usage of HMP or B &H as multiple testing correction at the second (between) layer, since similarities between the blocks can introduce a fairly strong positive correlation among the tests.

### Real data application

Here we illustrate our proposed testing approach on a yeast data set from [16], where both haplotype and allele frequencies have been obtained at 3 different time points. The experiment involves 4 haplotypes which are investigated on a grid of non-overlapping 30 KB windows. We applied the omnibus variant of the haplotype based test, as well as SNP based testing using the commonly used within-window Benjamini & Hochberg correction. For both methods, we also applied a Benjamini & Hochberg multiple testing correction across windows in order to control a false discovery rate of 0.05 at a genome-wide level. The three sequenced time points have been taken at cycles 0, 6 and 12, where each cycle is estimated to contain 15 to 20 generations. For our analysis, we take the mean and assume 17.5 generations per cycle which leads to 105 generations between adjacent sequenced time points.

We estimate the effective population size with the method proposed by [20], and use data from population 4k, at cycles 0 and 6. We did not include the frequencies at cycle 12 due to the high percentage of fixed or lost alleles, and the very low estimated effective population sizes, see Additional file 1: Section S.14 for more details. We also check windows with very highly correlated haplotypes by constructing UPGMA trees. Such windows may lead to false positive results due to multi-collinearity, when assuming that the error in haplotype reconstruction is negligible. However, we did not find this to be an issue for the windows we detect as selected.

Additionally, we note that the chi-squared test can become anti-conservative if the starting frequencies are too small, especially for a dataset that does not have intermediate time points. We also found that some of the starting allele frequencies are equal to zero, whilst increasing in frequency over time, or vice versa. Presumably, this is due to pool sequencing noise, but can still lead to biased testing results. To avoid such problems, we decided to remove any SNP or haplotype that has a corresponding starting frequency of less than 0.01, or larger than 0.99. Further, we excluded from our analysis a small number of SNPs with large allele frequency changes that could not be explained by underlying haplotype frequency changes.

Genome wide, the haplotype based test is able to detect selection in 658 windows whilst the SNP based test only in 47. Furthermore, all the 47 significant windows identified by the SNP based test are also identified by the haplotype based test. This is in line with our previous simulations that confirmed a higher power of haplotype based tests under most considered scenarios. See Additional file 1: Section S.15 for further details.

**Fig. 8:**
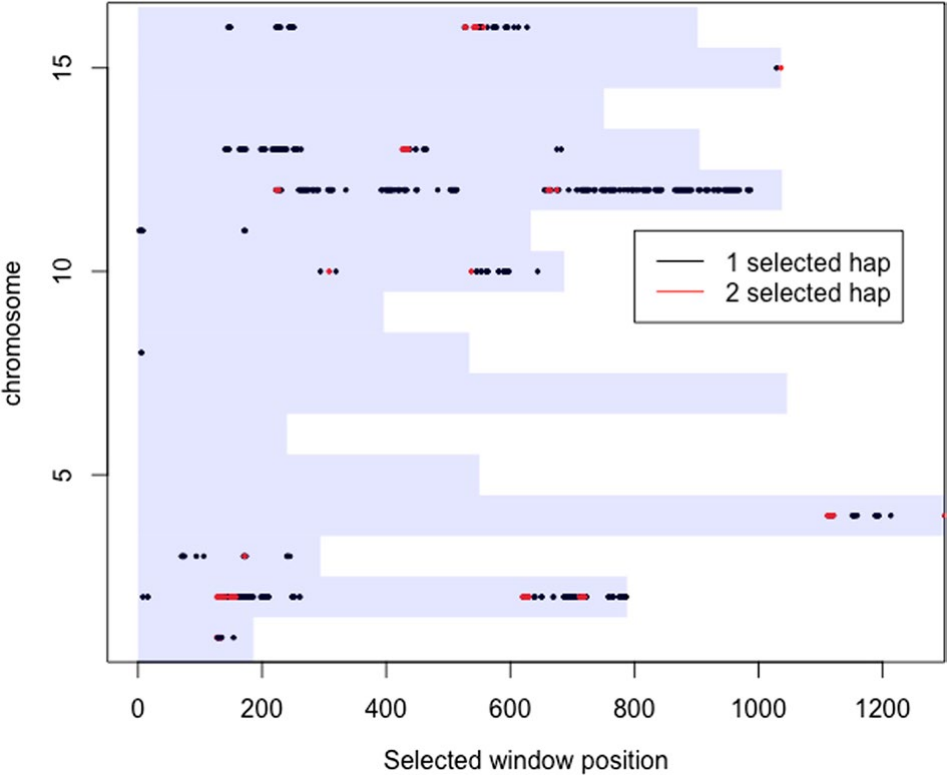
Position of selected windows according to haplotype based test. The background rectangles represent the chromosomes, and the positions represent the window positions and can be translated into positions on the genome when multiplying them by 30 KB. The dots represent positions of selected windows found by the (omnibus) haplotype based test, and the colours indicate the number of selected haplotypes inferred by our proposed post hoc test

Figure 8 displays a genome-wide summary of the selected window positions. To better understand the selective architecture, we also provide the estimated numbers of selected haplotypes. On chromosome 11, for instance, only one selected haplotype has been identified for all windows inferred as affected by selection. To better understand the relevance of our newly identified locations, one may look at the annotations provided by the Saccharomyces yeast Genome Database (SGD) [41]. As an example, windows 696 and 697 of chromosome 2 are both detected as selected by the haplotype based test but none by the SNP based test. According to SGD, these windows belong to the GPX2 gene which protects cells from phospholipid hydroperoxides and nonphospholipid peroxides during oxidative stress.

We also see that some selected windows cluster together which could point toward hitchhiking effects in neighbouring windows. Similar numbers of selected haplotypes for nearby windows, may also point towards selected haplotypes extending over multiple windows.

As a further example, we applied pairwise tests to the selected windows at chromosomes 1 and 11, and summarise the results in Table 3, with haplotypes H1, H2, H3, H4 denoting the haplotypes alt_YEE_hap_A1_00, alt_YEE_hap_A2_00, alt_YEE_hap_B3_00, alt_YEE_hap_B4_00 respectively.

**Table 3:** Proportion of windows for which the pairwise tests provide evidence of fitness differences between specific haplotype pairs

Chromosome	Haplotype pairs					
	H1-H2	H1-H3	H1-H4	H2-H3	H2-H4	H3-H4
1	0.4	1	0.1	1	0.4	1
11	0.1	1	0	1	0	1

In Chromosome 11 we only detected windows with 1 selected haplotype. The pairwise test supports this theory, showing H3 being the only haplotype that has a significant fitness difference against all others. Using this knowledge, we can assume that H3 is the selected haplotype at these selected windows. Chromosome 1 on the other hand, has both windows with one and two selected haplotypes according to our previous tests. Again, we see that for all selected windows, H3 differs in fitness, while H2 is the other significant haplotype in fewer than half of the windows. We can therefore infer that H3 is again always selected, but H2 is also selected for some of the windows which confirms our previous testing results.

## Discussion

It is well known that haplotypes harbour valuable information that can be helpful for the understanding of the selective architecture [11]. In this work, we show that haplotype based tests can effectively detect selection in genomic regions under a wide range of evolve and resequence scenarios. Furthermore, our proposed post-hoc tools are meant to identify the number of selected haplotypes and to investigate fitness differences between haplotypes. This helps to better understand features of the selective architecture such as polygenic adaptation. The post hoc tests perform especially well when replicate populations show consistent signals.

In order to apply haplotype based tests, we focus on relatively short genomic windows such that the haplotypes are not much affected by recombination and their frequency changes can be followed over time. As a guideline, [14] provides the window size for various organisms such that the impact of recombination can be assumed negligible. These loci with limited recombination have also been referred to as microhaplotypes [42], and are considered as a new type of genetic marker. In most situations, our haplotype based test has more power than the SNP based one.

For experimental designs involving too many haplotypes, we propose methods of collapsing their frequencies. This significantly improves the performance of our method since the starting haplotype frequencies increase when lowering the number of haplotypes. These methods either use SNPs identified by preliminary tests or a haplotype block based approach. They help to reduce the loss in power incurred with an increasing number of haplotypes and increase the range of scenarios for which haplotype based tests are a good choice. In experiments involving large numbers of haplotypes ($\ge$ 100), approaches where the results based on haplotypes and SNPs are combined would be an interesting follow-up.

## Conclusion

We studied our proposed haplotype based test under a wide range of simulated experiments. We think that our investigation of the effect of different design parameters on the power will be helpful for planning new experiments. The variables that influence the performance of the test most are the number of haplotypes and of selected haplotypes. These parameters also affect the power of the SNP based tests. The size of the considered window is also relevant for SNP based tests, as it influences the amount of required multiple testing correction. However, it does not affect the power of haplotype based tests, as long as the number of involved haplotypes does not become too large.

In this paper, most of our simulation results were obtained for scenarios with known SNP and haplotype frequencies. However, often the haplotype frequencies are unknown in applications [34, 43]. Thus we also provide versions of our tests for scenarios with unknown or partially unknown haplotype frequencies. With sampling errors, the relative performance between haplotype and SNP based tests remains similar to the known frequency scenario. The advantage of haplotype based tests can increase considerably, however, if additional noise is introduced by pool sequencing. Since haplotype frequencies can be estimated by combining information across many SNPs using for instance regression, haplotype based methods are much less affected by pool sequencing noise than approaches focusing on individual SNPs.

Our results demonstrate that haplotype based tests for selection provide attractive tools to better understand selective architectures in the context of experimental evolution. Both the initial and the post hoc tests provide useful tools for this purpose.

### Supplementary Information

**Additional file 1.** Supplementary Material.

## Data Availability

We implemented our proposed approach in an R [44] package available at https://github.com/xthchen/haplotest. The datasets used are from [34] and in [16].

## Abbreviations

SNP: Single nucleotide polymorphism
E&R: Evolve and resequence
HMP: Harmonic mean *p* value
B&H: Benjamini & Hochberg
CMH: Cochran–Mantel–Haenszel
ROC: Receiver operating characteristic
AUC: Area under curve
$N_e$: Effective population size
GWAS: Genome-wide association studies
