# Supplementary material for: Haplotype based testing for a better understanding of the selective architecture
Source: BMC Bioinformatics. 2023 Aug 26;24:322. doi: 10.1186/s12859-023-05437-3 (PMC10463365; doi:10.1186/s12859-023-05437-3)
Supplement: Supplementary file 1 — Additional file 1. Supplementary Material. [file 12859_2023_5437_MOESM1_ESM.pdf]

Supplementary material

S.1 Multiple testing corrections

We have considered using other multiple testing corrections for our haplotype based approach, such as the commonly used Bonferroni [1] and Benjamini & Hochberg [2], as well as Vovk's methods proposed in [3]. During our preliminary work however, these tests have shown to be much more conservative than omnibus and HMP due to stricter constraints.

S.1.1 Vovk's methods

[3] proposed combination methods that promise type I error control under any dependencies given that the number of p-values is large. The methods are extremely conservative similar to the Bonferroni correction, which is a special case of the proposed methods, but can be useful if having very few false positives is the goal. The methods depend on a parameter value r, with guidelines suggesting that more dependent p-values should use larger values of r. The merging function dependent on the total number of p-values N and parameter r is defined as follows:

$$M_{r,N} = \begin{cases} \left(\prod_{i=1}^{N} p_i\right)^{\frac{1}{N}}, & r = 0 \\ \left(\sum_{i=1}^{N} p_i^r / N\right)^{\frac{1}{N}}, & \text{otherwise} \end{cases} \tag{25}$$

with evidence of rejecting the null hypothesis present if:

$$\alpha > \begin{cases} (r+1)^{\frac{1}{r}} M_{r,N}, & r \in (-1, \infty] \\ \ln N^e M_{r,N}, & r = -1 \\ \frac{r}{r+1} N^{1+1/r} M_{r,N}, & r \in [-\infty, -1) \end{cases} \tag{26}$$

The Bonferroni correction would correspond to the case when $r = -\infty$.

S.2 Test statistic under different scenarios

In the paper we have shown the test statistic for haplotype based testing in a scenario where haplotype frequencies are known, and effective population size does not change over time. This however, might not be realistic. Usually, haplotype and allele frequencies are estimated with either or both sampling and pool sequencing noises. When such is the case, variances from these noises need to be taken into account to avoid type I error violations. Here, we provide test statistics for a wider range of scenarios, where the frequencies are estimated with different sources of variance.

S.2.1 Test statistic when all haplotype frequencies are estimated with sampling variance

Assuming a sample size of size $n_t^{(r)}$ is used to estimate the haplotype frequencies at time t and replicate r, an additional variance component of frequency estimation is present when compared to the case with known frequencies.

$$T_{\text{CMH}} = \frac{\sum_{r=1}^{R} n_0^{(r)^2} n_T^{(r)^2} \left(f_{j,0}^{(r)} - f_{j,T}^{(r)}\right)^2}{\sum_{r=1}^{R} n_T^{(r)^2} \hat{s}_{j,0}^{(r)^2} + n_0^{(r)^2} \hat{s}_{j,T}^{(r)^2}} \tag{27}$$

with $\left(\hat{s}_{j,0}^{(r)}\right)^2 / n_0^{(r)}$ and $\left(\hat{s}_{j,T}^{(r)}\right)^2 / n_0^{(T)}$ being consistent estimators of $Var(f_{j,0}^{(r)})$ and $Var(f_{j,T}^{(r)})$ respectively:

$$\left(\hat{s}_{j,0}^{(r)}\right)^2 = \hat{f}_{j,0}^{(r)}(1 - \hat{f}_{j,0}^{(r)}) n_0^{(r)} \tag{28}$$

$$\left(\hat{s}_{j,T}^{(r)}\right)^2 = n_T^{(r)} \left(\hat{f}_{j,T}^{(r)}(1 - \hat{f}_{j,T}^{(r)}) + (n_T^{(r)} - 1)\sigma_{\text{drift}}^{(r)}\right) \tag{29}$$

S.2.2 Test statistic when starting haplotype frequencies are known, others estimated with sampling variance

Similar to the scenario in Section S.2.1, except the variance component for starting frequencies is nullified.

$$T_{\text{CMH}} = \frac{\sum_{r=1}^{R} N_e^{(r)^2} n_T^{(r)^2} \left(f_{j,0}^{(r)} - f_{j,T}^{(r)}\right)^2}{\sum_{r=1}^{R} N_e^{(r)^2} \hat{s}_{j,T}^{(r)^2}} \tag{30}$$

with $\left(\hat{s}_{j,T}^{(r)}\right)^2 / n_0^{(T)}$ being consistent estimators of $Var(f_{j,T}^{(r)})$:

$$\left(\hat{s}_{j,T}^{(r)}\right)^2 = n_T^{(r)} \left(\hat{f}_{j,T}^{(r)}(1 - \hat{f}_{j,T}^{(r)}) + (n_T^{(r)} - 1)\sigma_{\text{drift}}^{(r)}\right) \tag{31}$$

S.2.3 Test statistic when all frequencies are estimated with both sampling and pool-sequencing variances
Assuming a sample size of $n_t^{(r)}$ and coverage of $u_t^{(r)}$ at time t and replicate r, additional components of variance are needed to be taken into account. The new test statistic under this scenario is given as follows:

$$T_{\text{CMH}} = \frac{\sum_{r=1}^{R} N_e^{(r)2} u_T^{(r)2} \left(\tilde{f}_{j,0}^{(r)} - \tilde{f}_{j,T}^{(r)}\right)^2}{\sum_{r=1}^{R} u_T^{(r)2} \tilde{s}_{j,0}^{(r)2} + u_0^{(r)2} \tilde{s}_{j,T}^{(r)2}} \tag{32}$$

with $\left(\tilde{s}_{j,0}^{(r)}\right)^2 / u_0^{(r)}$ and $\left(\tilde{s}_{j,T}^{(r)}\right)^2 / u_0^{(T)}$ being consistent estimators of $Var(f_{j,0}^{(r)})$ and $Var(f_{j,T}^{(r)})$ respectively. For SNP based test, this can be computed by:

$$\left(\tilde{s}_{j,0}^{(r)}\right)^2 = \tilde{f}_{j,0}^{(r)}(1 - \tilde{f}_{j,0}^{(r)}) u_0^{(r)} \left(1 + \frac{u_0^{(r)} - 1}{n_0^{(r)}}\right) \tag{33}$$

$$\left(\tilde{s}_{j,T}^{(r)}\right)^2 = u_T^{(r)} \left(\tilde{f}_{j,T}^{(r)}(1 - \tilde{f}_{j,T}^{(r)}) \left(1 + \frac{u_T^{(r)} - 1}{n_T^{(r)}}\right) + \frac{(u_T^{(r)} - 1)(n_T^{(r)} - 1)}{n_T^{(r)}} \sigma_{\text{drift}}^{(r)}\right) \tag{34}$$

and for haplotype based test, these are the variances of the estimated regression coefficients $\hat{\mathbf{F}}_P^{(r)}$ at time points 0 and T. Further derivations for the SNP based tests can be found in [4].

S.2.4 Drift variance when N_e changes over time
If N_e changes due to experimental design, and is measured at all sequenced time points, or due to testing for number of selected haplotypes, the variance of drift with changing N_e, $\sigma_{drift,\Delta N_e}$ can be computed by:

$$\sigma_{drift,\Delta N_e} = \sum_{i=0}^{k-2} f_{i,t_i,1}(1 - f_{i,t_i,1}) \left(1 - \left(1 - \frac{N_{e_i}^{-1} + N_{e_{i+1}}^{-1}}{2}\right)^{t_{i+1} - t_i}\right) \tag{35}$$

where N_{e_i} is the N_e at timepoint t_i. $\sigma_{drift,\Delta N_e}$ can then be used in place of σ_{drift} in the previous test statistics.

S.2.5 Testing for the number of selected haplotypes when frequencies are estimated with sampling variance
Assuming the presence of R replicates and estimated frequencies, the m_1 haplotype that provides the maximum change in frequency outcome is calculated as follows:

$$m_1 : \max_{m_1} \sum_{r=1}^{R} \frac{\hat{f}_{m_1,T}^{(r)} - \hat{f}_{m_1,0}^{(r)}}{\sqrt{(\hat{s}_{m_1,T}^{(r)})^2 / n_T^{(r)} + (\hat{s}_{m_1,0}^{(r)})^2 / n_0^{(r)}}} \tag{36}$$

Notice that due to the noise introduced by sequencing and sampling, estimated intermediate frequencies can be zero, while latter frequencies deviate from zero again.

S.3 Combining haplotypes using SNP based test
As introduced in Section 2.5, by removing certain SNPs, it could be possible that several haplotypes now share the same set of nucleotides, and their frequencies can thus be combined. Even though due to hitchhiking, smaller p-values do not necessarily indicate a selected target, selected targets will unlikely result in large p-values. Thus through the removal of SNPs that give p-values higher than some certain threshold β, the combined haplotypes are likely to either be both targets or non-targets of selection, achieving the goal of haplotype number reduction while not diluting the original signal. Below we explain this idea in more detail.

Step 1: From allele frequency matrix $\mathbf{A}_{N_{SNP} \times (T+1)}$, apply adapted chi-square test [4] as in haplotype based test.
Step 2: Using the obtained p-values $\{p_i\}_{i \in [1, N_{SNP}]}$, create the filtered set of SNPs $\{h_{j,\cdot}\}_{p_j < \beta}$.
Step 3: Create the new haplotype structure matrix $\mathbf{H}^{filt}$ by removing all SNPs from $\mathbf{H}$ not present in the filtered SNP set.
Step 4: Combine all haplotype frequencies that now have the same nucleotide $f_k^{filt} = \sum_{l:h_{,l}^{filt} = h_{,k}^{filt}} f_l$.
Step 5: Apply the haplotype based test on the new combined haplotype frequency matrix $\mathbf{F}^{filt}$.

S.4 Combining haplotypes using haplotype blocks
In this section we provide more details about the computation of haplotype blocks. Suppose in a simple setup where we have 2 SNPs and hence 4 haplotypes, with the allele frequencies of the two SNPs being u, v respectively, we write the haplotype frequencies as $h_{1,1}, h_{1,0}, h_{0,1}, h_{0,0}$ dependent on the nucleotide they hold. The coefficient of linkage disequilibrium, D, is then calculated by:

$$D = h_{1,1} - uv \tag{37}$$

This value is not informative as its size is relative to the allele frequencies, therefore usually a normalised version D' proposed by Lewontin (1964) [5] is used to measure LD:

$$D' = \frac{D}{D_{\max}} \tag{38}$$

$$D_{\max} = \begin{cases} \max[-uv, -(1-u)(1-v)], & \text{if } D < 0. \\ \min[u(1-v), (1-u)v], & \text{if } D > 0. \end{cases} \tag{39}$$

The variance of D' can be estimated as suggested in [6]:

$$Var(\hat{D'}) \approx \frac{1}{n(D_{\max})^2}((1-|D'|)(nVar(D) - |D'|D_{\max}(ua + (1-u)b - 2|D|)) + |D'|x_i(1-x_i)) \tag{40}$$

$$x_i = \begin{cases} h_{1,1}, & \text{if } D_{\max} = -uv. \\ h_{1,0}, & \text{if } D_{\max} = u(1-v). \\ h_{0,1}, & \text{if } D_{\max} = (1-u)v. \\ h_{0,0}, & \text{if } D_{\max} = -(1-u)(1-v). \end{cases} \tag{41}$$

where n is the total number of haplotypes of the entire interested SNP region, and $a = v$, $b = (1-v)$ if $D' < 0$ and vice versa. If we write the normalised coefficient of linkage disequilibrium between some SNP pairs i and j as $D'_{i,j}$, by using this variance approximation and assuming $D'_{i,j}$ to be normally distributed, we can calculate its 90% confidence interval, with the upper and lower intervals being $U_{i,j}$ and $L_{i,j}$ respectively. The definition of an haplotype block is then given by Gabriel et al. (2002) [7], where the pair of SNPs are said to be in strong linkage disequilibrium (LD) if $L_{i,j} \geq 0.7$ and $U_{i,j} \geq 0.98$ and the pair shows strong evidence of historical recombination, or EHR if $U_{i,j} < 0.9$.
A haplotype block from SNP i to SNP j can only exist if the SNP pairs i and j are in strong LD. The number of SNP pairs that are in strong LD within the block needs to be at least 19 times the number of SNP pairs that are in EHR. After finding all possible haplotype block within the given window, we rank the haplotype blocks according to SNP length, and select non-overlapping blocks as our block choice starting from the highest rank. We only use haplotype blocks that extend over more than 2 SNPs, as little information is gained from very short blocks.

S.5 Type I error control without replicates
In Figure S1 we show that there can be type I error control issues, when no replicates are present, and the number of founder haplotypes are very small. This issue however is solved if there are more founder haplotypes, or if any replicate population is present.

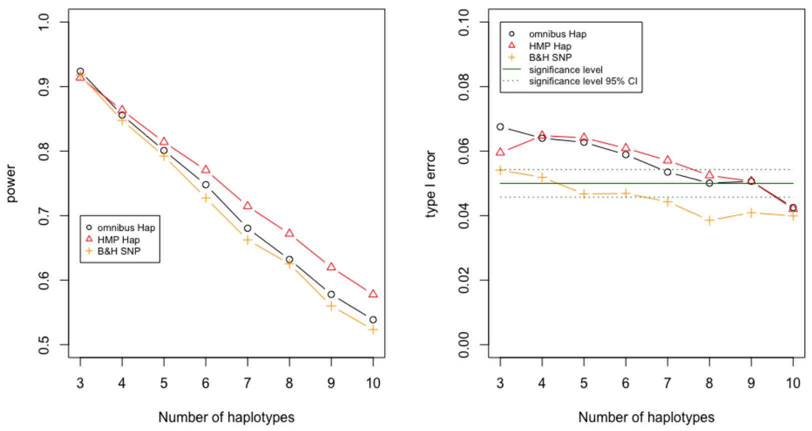

Figure S1: **Results when changing the number of total haplotypes with no replicate population.** Power and Type I error curves are plotted for both haplotype and SNP based test under various N_{Hap}, no replicate population, with all other parameters default as outlined in Table 2.

S.6 Effect on power and AUC when changing model parameters

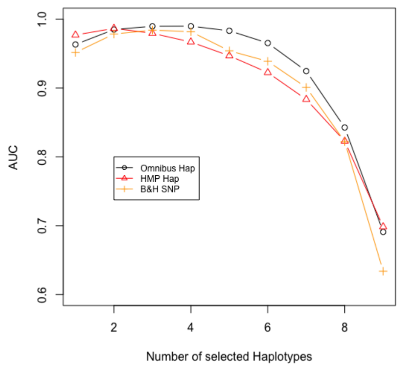

Figure S2: **Results when changing the number of selected haplotypes h_{Sel}.** Area under curve (AUC) of the ROC curves are provided for the haplotype and SNP based tests under various values of h_{Sel}, with all other parameters default as outlined in Table 2.

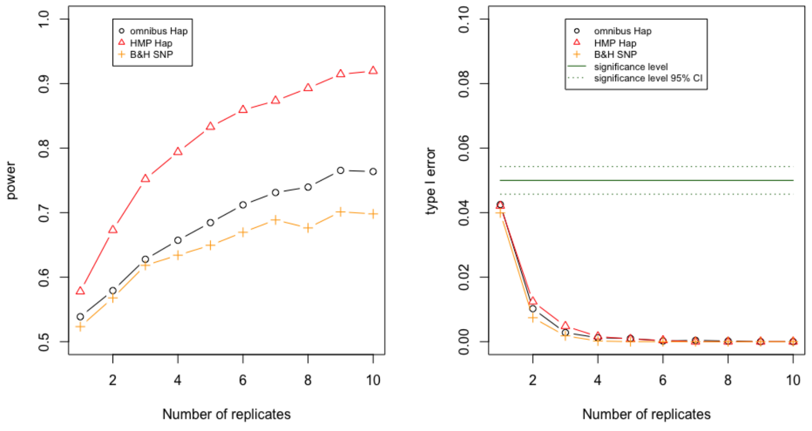

Figure S3: **Results when changing the number of replicate populations.** Power and Type I error curves are plotted for both haplotype and SNP based test under various values for the number of replicates R, with all other parameters default as outlined in Table 2.

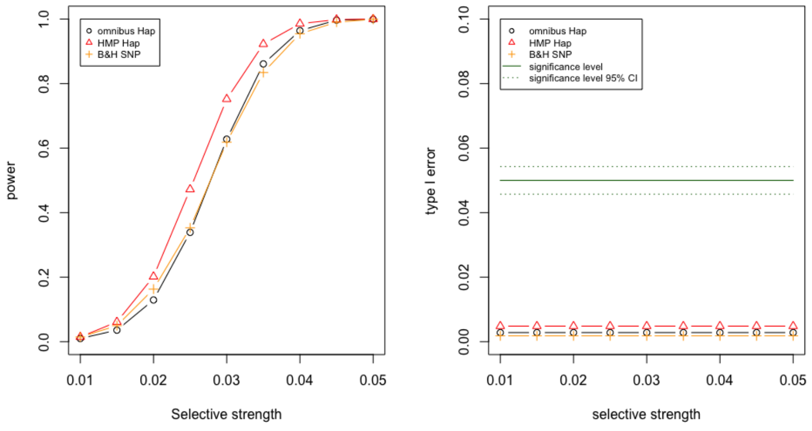

Figure S4: **Results when changing selection strength.** Power and Type I error curves are plotted for both haplotype and SNP based test under various values for the selection coefficient s, with all other parameters default as outlined in Table 2.

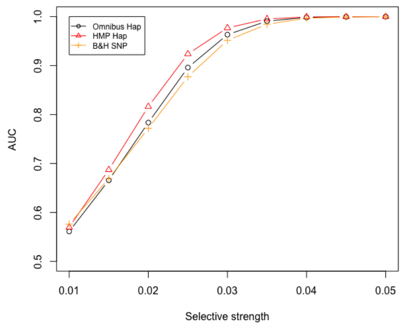

Figure S5: **Results when changing selection strength.** AUC of the ROC curves are plotted for both haplotype and SNP based test under various values for the selection coefficient s, with all other parameters default as outlined in Table 2.

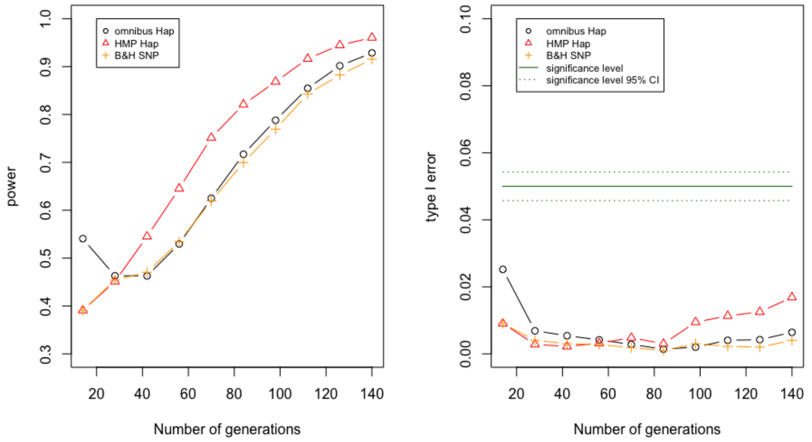

Figure S6: **Results when changing the total number of generations.** Power and Type I error curves are plotted for both haplotype and SNP based test under various total number of generations, with all other parameters default as outlined in Table 2.

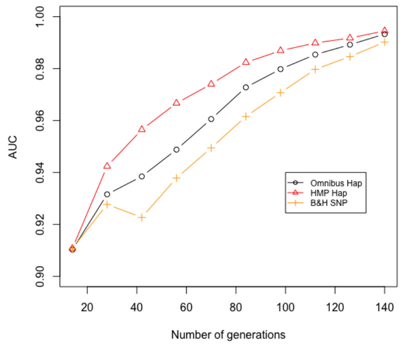

Figure S7: **Results when changing the total number of generations.** AUC of the ROC curves are plotted for both haplotype and SNP based test under various total number of generations, with all other parameters default as outlined in Table 2.

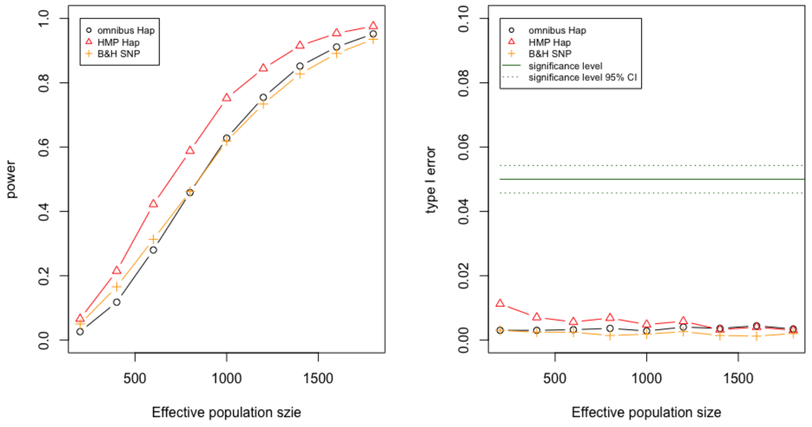

Figure S8: **Results when changing effective population size.** Power and Type I error curves are plotted for both haplotype and SNP based test under various N_e, with all other parameters default as outlined in Table 2.

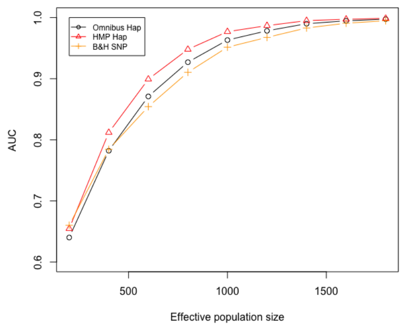

Figure S9: **Results when changing effective population size.** AUC of the ROC curves are plotted for both haplotype and SNP based test under various N_e, with all other parameters default as outlined in Table 2.

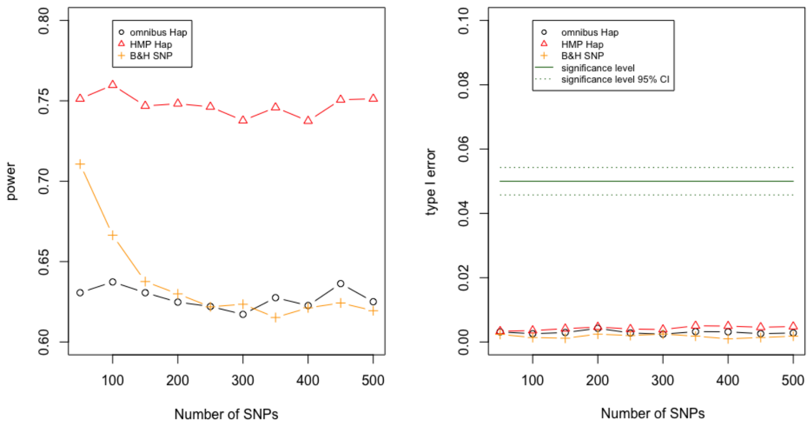

Figure S10: **Results when changing the number of SNPs.** Power and Type I error curves are plotted for both haplotype and SNP based test under various N_{SNP}, with all other parameters default as outlined in Table 2.

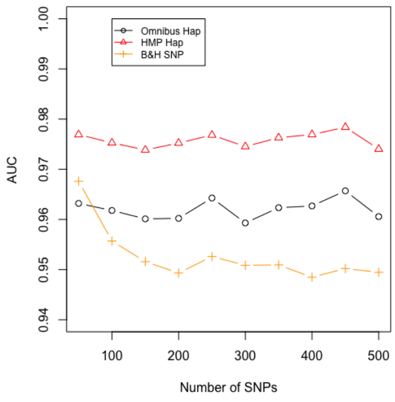

Figure S11: **Results when changing the number of SNPs.** AUC of the ROC curves are plotted for both haplotype and SNP based test under various N_{SNP}, with all other parameters default as outlined in Table 2.

S.7 Different scenarios of frequency estimation

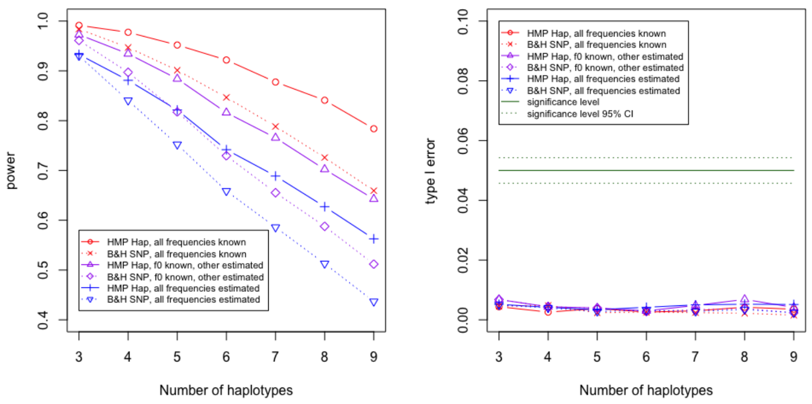

Figure S12: **Results when changing the number of total haplotypes under 3 scenarios.** Power and Type I error curves are plotted for both haplotype and SNP based test under various N_{Hap}, with all other parameters default as outlined in Table 2 under 3 scenarios. Scenario 1: all haplotype frequencies are known. Scenario 2: starting haplotype frequencies are known and frequencies for later time points are estimated with a sample size of 80. Scenario 3: all haplotype frequencies are estimated with a sample size of 80.

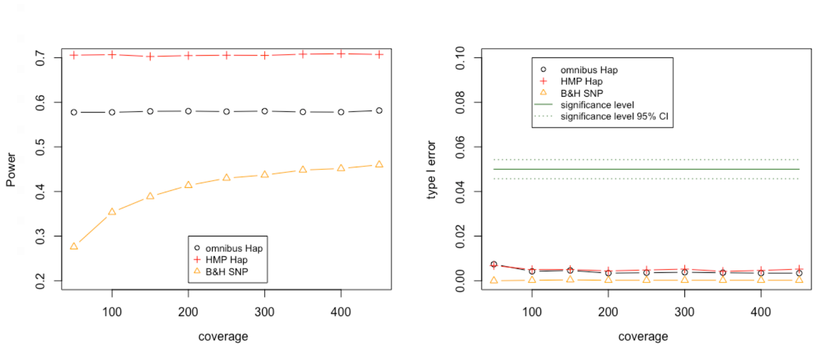

Figure S13: **Results when changing the coverage.** Power and type I error curves are plotted for haplotype and SNP based test under various sampling size. All frequencies are estimated with a sample size of 500, with other parameters default as outlined in Table 2.

S.8 Further results on testing for the number of selected haplotypes

Here we show a wider range of scenarios where we perform the post hoc test for the number of selected haplotypes. We show results when using omnibus and HMP as multiple testing corrections and consider scenarios with and without replicate in Figures S14, S15 and S16.

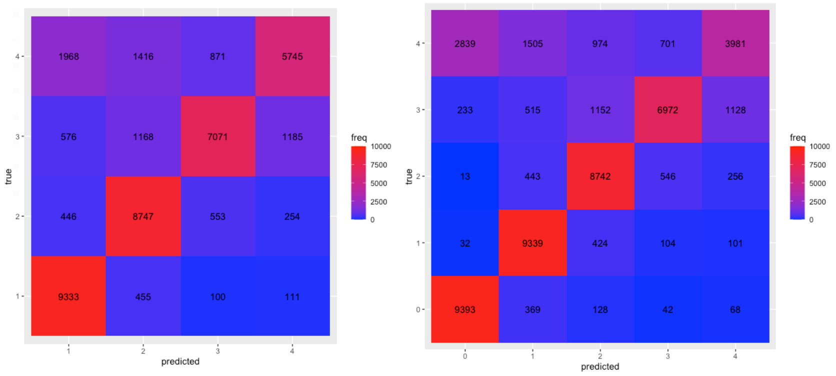

Figure S14: **Result showing the predictability of haplotype based iterative testing.** Omnibus is used for multiple testing correction under all scenarios. The heat map is simulated using 5 founder haplotypes and a selection strength of $s = 0.05$, 1 replicate population, and all other parameters default as outlined in Table 2. In the left panel, the test is conditional on the presence of selection. In the right panel, the test is unconditional.

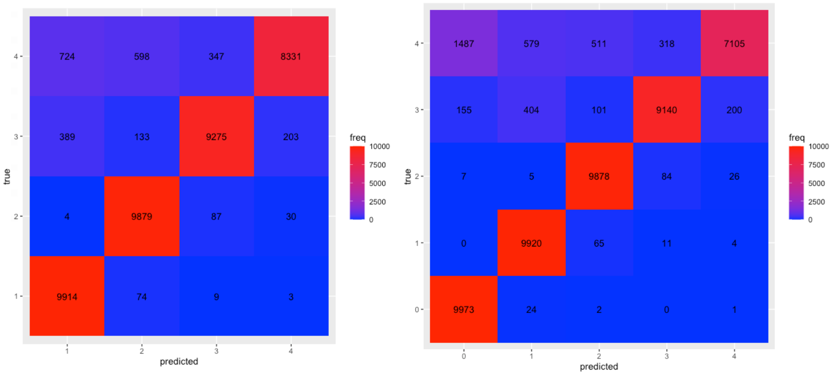

Figure S15: **Results showing the prediction accuracy of haplotype based iterative testing.** The HMP method is used for multiple testing correction in all scenarios. The heat map has been obtained using 5 founder haplotypes and a selection strength of $s = 0.05$, and all other parameters default as outlined in Table 2. In the left panel, the test is conditional on the presence of selection. In the right panel, the test is unconditional.

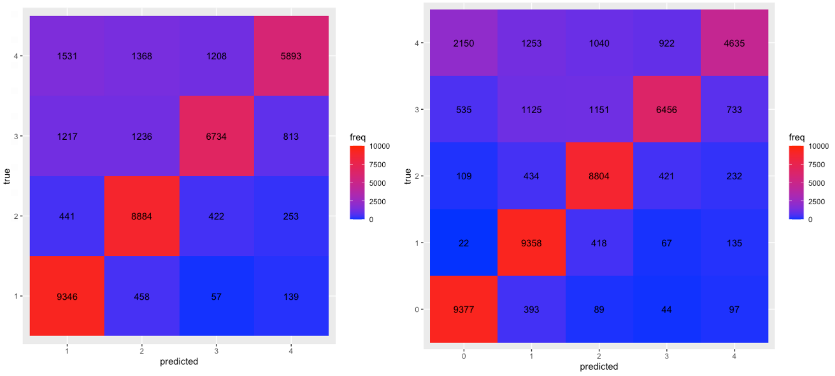

Figure S16: **Result showing the predictability of haplotype based iterative testing.** HMP is used for multiple testing correction under all scenarios. The heat map is simulated using 5 founder haplotypes and a selection strength of $s = 0.05$, 1 replicate population, with all other parameters default as outlined in Table 2. In the left panel, the test is conditional on the presence of selection. In the right panel, the test is unconditional.

S.9 Type I error of testing for the number of selected haplotypes under low selective strength

Figure S17 shows a scenario where we observed violations in the conditional type I error for our proposed post hoc test. However, as shown below, the unconditional type I error remains under control.

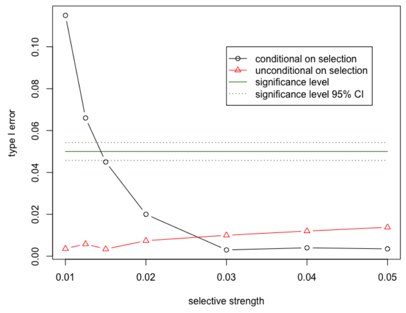

Figure S17: **Type I error plot when testing for one vs more than one selected haplotype.** The type I errors were computed either conditional or unconditional on the rejection of the initial test for selection. The omnibus test is used for multiple testing correction. Data is simulated using 5 founder haplotypes and different selection coefficients s, with all other parameters as outlined in Table 2.

S.10 Further results on pairwise test

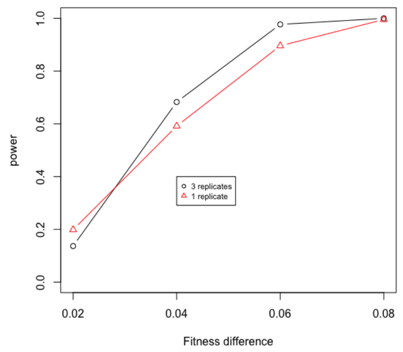

Figure S18: **Results for pairwise tests when all haplotypes have different fitness.** B&H is used for multiple testing correction. Data is simulated using 5 founder haplotypes, each having a fitness of (1,1.02,1.04,1.06,1.08), 1 or 3 replicates, with all other parameters default as outlined in Table 2.

The availability of replicate populations leads to more power for most scenarios. However it under-performs when the fitness difference is only 0.02. We notice a similar issue with replicate populations when applying our haplotype based test on cases where selected haplotypes have a very small selective advantage against others, see Section S.11. This is due to the adapted CMH test being more conservative than the adapted chi-squared test. If the selective strength is high enough, the information provided by the additional replicates makes the CMH test more powerful despite its conservativeness. When there is little signal (fitness difference 0.02), it leads to the lower power seen in both mentioned figures.

Since all fitness values are different to each other in this scenario, it is not possible to analyse type I error. Thus, we simulated one additional scenario where we randomly set two of the fitness values to 1 at each iteration, and observed that type I error is under control.

S.11 Comparing chi-squared and CMH test under low selective strength

In Section S.10 we observed that the pairwise test with the adapted CMH test has lower power than with the adapted chi-squared test at very low fitness differences, despite the increase in replicates. We show in Figure S19 that, this also apply more generally to non-pairwise test scenarios. Counter-intuitively, here we observe that at very low selective strength, increasing the number of replicates resulted in lower power when applying the adapted CMH test. This is caused by the CMH test being much more conservative than the adapted chi-squared test. In Figure

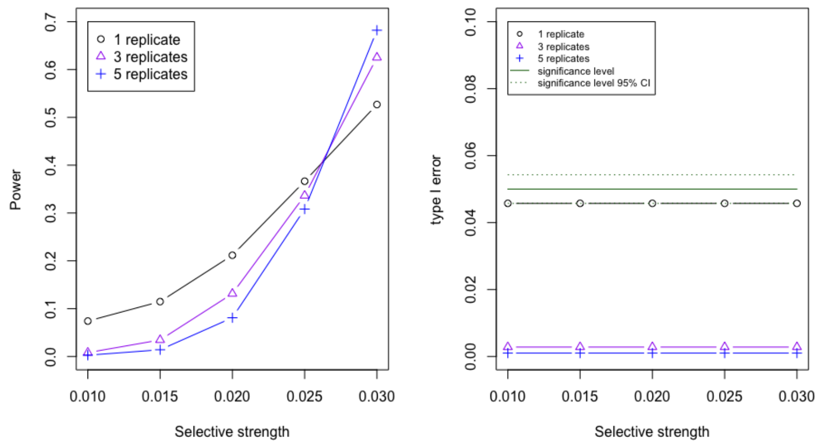

Figure S19: **Results when changing selection strength and number of replicate populations.** Power and type I error curves are plotted for haplotype based test using omnibus as p-value combination method under various s and R, with all other parameters default as outlined in Table 2.

S20 we show an additional scenario where we apply the adapted chi-squared test separately on each of the three replicates, and combine their results using a Benjamini & Hochberg multiple testing correction. Here we see that this method provides a significant increase in terms of power when compared to the scenario with one replicate, but also a much higher type I error when compared to the CMH test. We also plotted ROC curves in Figure S21 and show that even at 0.02 selective strength, despite the low power and conservativeness, CMH test is still the best performing test overall for haplotype frequencies, in terms of AUC.

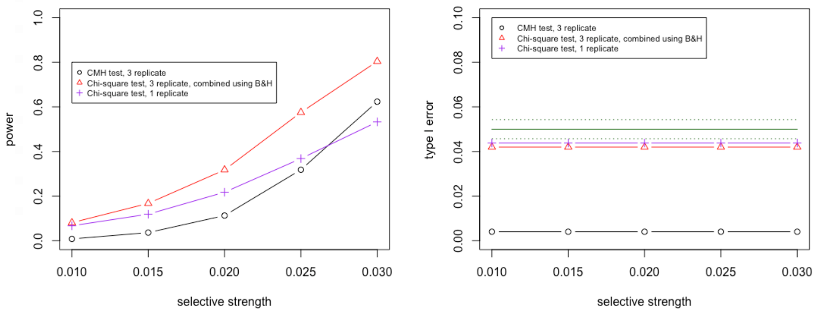

Figure S20: **Results when changing selection strength, number of replicate, using 2 testing methods.** Power and type I error curves are plotted for haplotype based test using omnibus as p-value combination method under various s and R, with all other parameters default as outlined in Table 2.

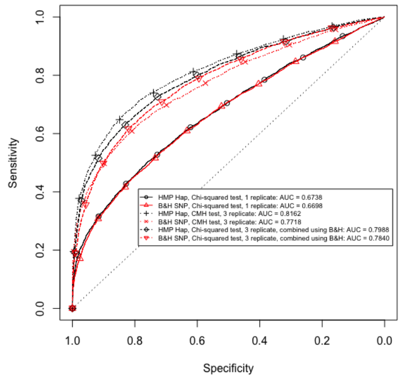

Figure S21: **Results when changing the number of replicate and testing methods.** ROC curves of haplotype and SNP based test when changing the testing method and number of replicates. The selective strength is set to be 0.02, with other parameters default as outlined in Table 2.

S.12 Further results for combining haplotypes

In this section we provide additional results for the proposed methods to combine haplotypes when the total number of haplotypes is too large. Figure S22 shows the ROC curves for the original haplotype and SNP based tests for selection and the tests where the SNP and block based methods for combining the haplotypes are used. Figures S23 and S24 compare the methods when changing the total number of haplotypes with respect to type I error and AUC. Figure S25 explores a special scenario where only 1 haplotype is selected despite a large number of founder haplotypes. Figure S26 shows the effect of changing inclusion threshold of the combination methods.

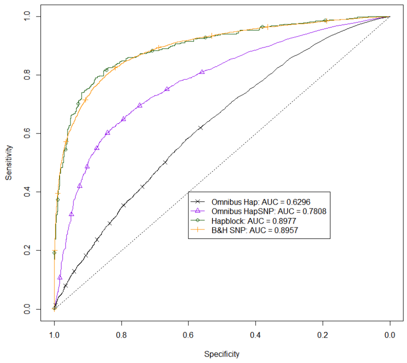

Figure S22: **ROC curves for different testing method with 100 haplotypes.** ROC curves of haplotype, HapSNP, hapblock and SNP based tests are shown for 100 total haplotypes, 50 selected haplotypes and no replicate population with all other parameters default as outlined in Table 2. Hapblock refers to the method of using haplotype blocks to reduce haplotype number outlined in Section 2.5.

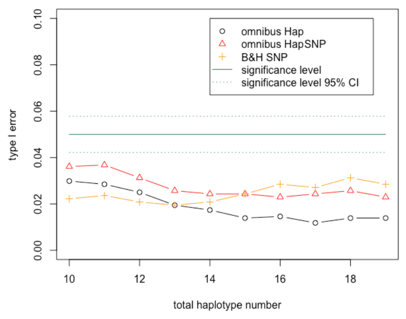

Figure S23: **Type I error for different values for the number of haplotypes N_{Hap}.** Type I error curve is plotted for the original haplotype, HapSNP, and SNP based test under various choices of N_{Hap}. HapSNP refers to the method of using the SNP based test to reduce the haplotype number prior to haplotype based testing outlined in Section 2.5. Here, the number of selected haplotypes is set to $h_{\text{Sel}} = N_{\text{Hap}}/2$. All other parameter values can be found in Table 2.

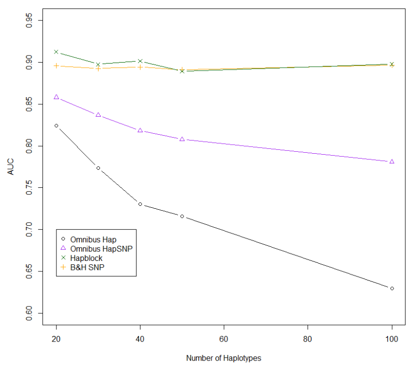

Figure S24: **AUC for different values for the number of haplotypes N_{Hap}.** AUC of the ROC curves are plotted for haplotype, HapSNP, Hapblock and SNP based test under various N_{Hap}. Here, the number of selected haplotypes is set to be half of the total haplotype number $h_{\text{Sel}} = N_{\text{Hap}}/2$, no replicate population, with all other parameters default as outlined in Table 2.

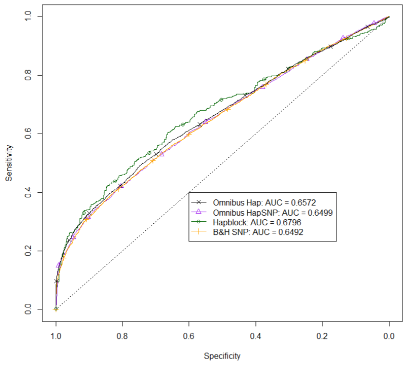

Figure S25: **ROC curves when there are 30 haplotypes, one of them selected.** Haplotype, HapSNP, Hapblock and SNP based tests are considered for 30 haplotypes, 1 selected haplotype and no replicate population, with all other parameters default as outlined in Table 2.

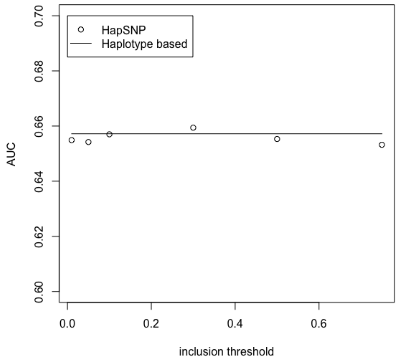

Figure S26: **AUC when changing the inclusion threshold of SNPs from the HapSNP procedure.** AUC for the HapSNP test is plotted under various inclusion threshold, compared with the AUC with haplotype based test, under parameters identical to Figure S25.

S.13 Scenarios when the haplotype starting frequencies are not equal

In all other simulations, we assume that the haplotype starting frequencies are always equal. Since this might not be the case in reality, we want to explore the scenarios of unequal starting frequency. To illustrate this, we plot the differences between AUC of ROCs for haplotype and SNP based tests. Additionally, all plots shown assume no replicate populations. From supplement Section S.6 we know that this would a more disadvantageous scenario for haplotype compared with the SNP based test.

We first explore the scenario of unequal starting frequencies with 3 founder haplotypes. Figure S27 and S28 show the case where there are 1 or 2 selected haplotypes respectively. We see that the haplotype based test consistently outperforms the SNP based test under almost all scenarios. In the few parameter sets where SNP based test outperforms the haplotype based test the AUC difference between the two tests was not significant. It is therefore likely that these disadvantageous scenarios are simply due to noise.

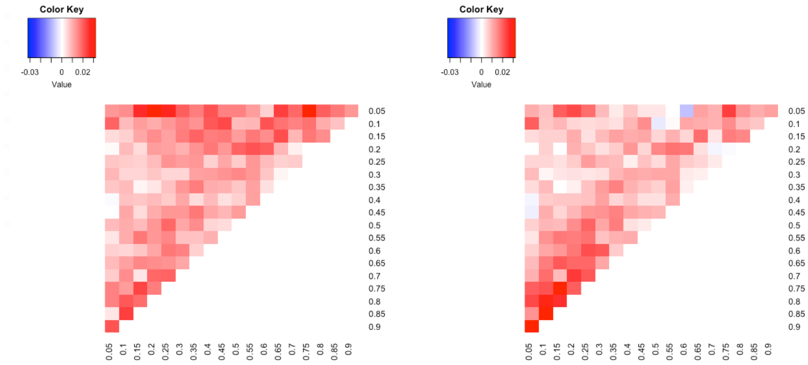

Figure S27: **Results when there is 1 selected haplotype.** The AUC of haplotype minus SNP based test is plotted under various starting haplotype frequencies. The frequency on the y-axis refers to the selected haplotype. HMP multiple testing correction is used in the left panel and the omnibus multiple testing correction in the right panel. Simulations are performed with 3 founders and 1 selected haplotype. The other parameters are as outlined in Table 2.

We next explore a scenario with 4 founder haplotypes. When one haplotype is selected (Figure S29), the results are similar to the scenario with 3 founder haplotypes (Figure S27) where the haplotype based test consistently performs better.

We note in Figure S30 that when there are 4 founder haplotypes and 2 selected haplotypes however, there are some scenarios where SNP based test is performing slightly better (blue dots). Whilst with replicates, haplotype based test might perform better at these scenarios, we want to explore whether these slightly worse performance can simply be regarded as noise. In supplement Figure S31, we compute the 95% confidence interval of the AUC differences, and choose to only plot the parameter sets that provide a significant AUC difference. We note that all scenarios where the haplotype based test is performing worse can potentially just be noise.

With 3 selected haplotypes shown in Figure S32, there is again little to no combination of starting frequencies where SNP based test is performing better, and similar to the other scenarios, the haplotype based test obtains the largest advantage whenever the combined frequency of the selected haplotypes is large.

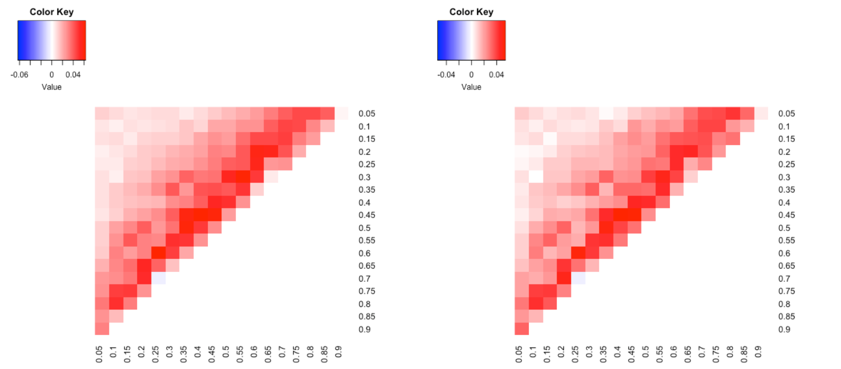

Figure S28: **Results when there are 2 selected haplotypes.** The AUC of haplotype minus SNP based test is plotted under various starting haplotype frequencies. The frequency on both the y and x-axis are always for the selected haplotypes. The Figure on the left panel is using HMP as multiple testing correction, while the Figure on the right panel is using omnibus. Simulations are performed with 3 founder and 2 selected haplotypes. The other parameters are as outlined in Table 2.

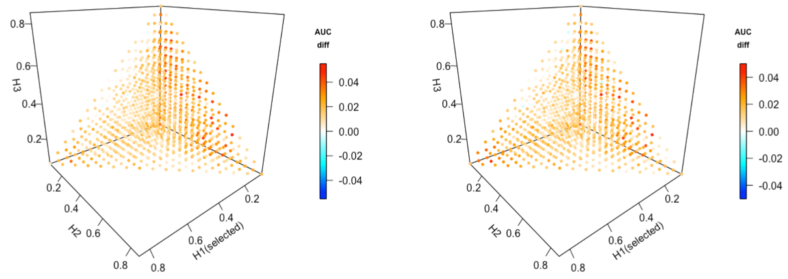

Figure S29: **Results when there is 1 selected haplotype.** The AUC of haplotype minus SNP based test is plotted under various starting haplotype frequencies. The Figure on the left panel is using HMP as multiple testing correction, while the Figure on the right panel is using omnibus. Simulations are performed with 4 founders and one selected haplotype. Here only 1,000 simulation is performed on each parameter set. The other parameters are as outlined in Table 2.

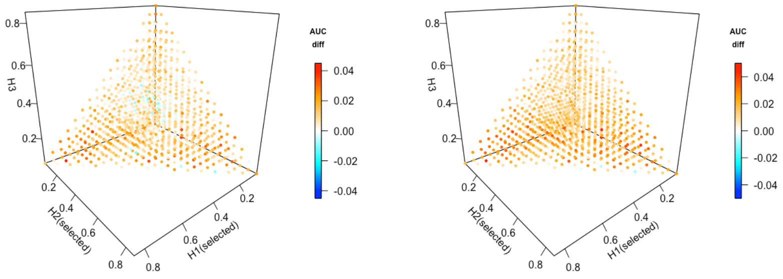

Figure S30: **Results when there are 2 selected haplotypes.** The AUC of haplotype minus SNP based test is plotted under various starting haplotype frequencies. The Figure on the left panel is using HMP as multiple testing correction, while the Figure on the right panel is using omnibus. Simulations are performed with 4 founder and 2 selected haplotypes. Here only 1,000 simulation is performed on each parameter set. The other parameters are as outlined in Table 2.

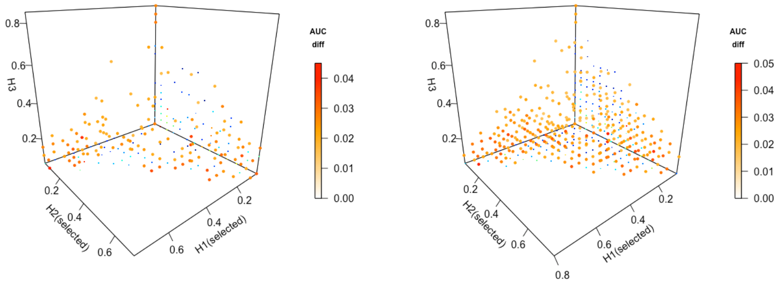

Figure S31: **Results when there are 2 selected haplotypes.** The AUC of haplotype minus SNP based test is plotted under various starting haplotype frequencies. We only plot the parameter sets giving results outside the 95% confidence interval of the AUC differences. The Figure on the left panel is using HMP as multiple testing correction, while the Figure on the right panel is using omnibus. Simulations are performed with 4 founder and 2 selected haplotypes. Here only 1,000 simulations is performed on each parameter set. The other parameters are as outlined in Table 2.

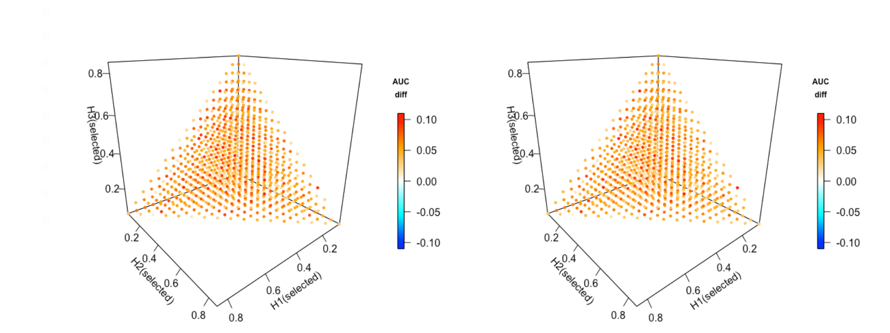

Figure S32: **Results when there are 3 selected haplotypes.** The AUC of haplotype minus SNP based test is plotted under various starting haplotype frequencies. The Figure on the left panel is using HMP as multiple testing correction, while the Figure on the right panel is using omnibus. Simulations are performed with 4 founder and 3 selected haplotypes. Here only 1,000 simulation is performed on each parameter set. The other parameters are as outlined in Table 2.

S.14 Justification of not using cycle 12 in Section 3.2

Wen analysing the real data from [8], we noticed an exceptionally high percentage of SNPs either fixed or lost at cycle 12 for population 4k, as shown in the table S1 below:

Chromosome	1	2	3	4	5	6	7	8	9	10	11	12	13	14	15	16
Percentage	51	64	56	58	46	58	65	72	67	61	51	59	60	57	70	70

Table S1: Percentage of SNPs either fixed or lost at last time point

This biases the SNP based test, as the fixation or loss of an allele can happen any time between cycle 6 and cycle 12, which would be roughly 105 generations. As no information is available between the cycles, we will not be able to obtain an accurate result using these data with the considered methods.
Moreover, the amount of average absolute frequency change between the first and second time point is much smaller than the frequency change between the second and third time point (Table S2).

Cycles \ Chromosomes	1	2	3	4	5	6	7	8	9	10	11	12	13	14	15	16
$0 \sim 6$	0.13	0.16	0.07	0.17	0.08	0.24	0.21	0.14	0.21	0.11	0.12	0.10	0.12	0.11	0.21	0.12
$6 \sim 12$	0.35	0.48	0.24	0.37	0.32	0.30	0.35	0.38	0.24	0.31	0.36	0.38	0.40	0.33	0.35	0.27

Table S2: Average absolute frequency change at each chromosome, between different cycles

This might be explained by a shrinking population size, and thus much higher drift at latter cycles. Indeed, using the N_e estimates proposed by [9], we observe a much lower median N_e estimate between the latter cycles, which makes the frequency changes noisier. Table S3 shows that it becomes therefore harder to separate selection and drift.

Chromosomes / Estimated N_e	1	2	3	4	5	6	7	8	9	10	11	12	13	14	15	16
$0 \sim 6$	1731	741	8611	950	4106	308	790	1496	641	2508	1905	4155	1819	2900	336	1162
$6 \sim 12$	1630	74	555	127	82	137	101	68	242	215	639	86	313	101	117	138

Table S3: Median of estimated N_e using allele frequencies between different cycles

S.15 Number of windows where selection is detected in real data application

Chromosomes	Total	1	2	3	4	5	6	7	8	9	10	11	12	13	14	15	16
Hap based	658	10	151	17	38	0	0	0	2	0	26	10	261	85	0	2	56
SNP based	47	0	0	0	0	0	0	0	0	0	0	0	47	0	0	0	0
Total window number	11482	186	788	294	1496	550	240	1046	534	396	687	633	1038	905	751	1036	902

Table S4: Absolute number of windows each method is able to detect as selected for each chromosome.

References

1. Wiberg RAW, Gaggiotti OE, Morrissey MB, Ritchie MG. Identifying consistent allele frequency differences in studies of stratified populations. Methods in Ecology and Evolution. 2017;8(12):1899–1909. Available from: https://doi.org/10.1111/2041-210X.12810.
2. Benjamini Y, Hochberg Y. Controlling the False Discovery Rate: A Practical and Powerful Approach to Multiple Testing. Journal of the Royal Statistical Society: Series B (Methodological). 1995;57(1):289–300. Available from: https://doi.org/10.1111/j.2517-6161.1995.tb02031.x.
3. Vovk V, Wang R. Combining p-values via averaging. Biometrika. 2020 06;107(4):791–808. Available from: https://doi.org/10.1093/biomet/asaa027.
4. Spitzer K, Pelizzola M, Futschik A. Modifying the Chi-square and the CMH test for population genetic inference: Adapting to overdispersion. The Annals of Applied Statistics. 2020;14(1):202 – 220. Available from: https://doi.org/10.1214/19-AOAS1301.
5. Lewontin RC. THE INTERACTION OF SELECTION AND LINKAGE. I. GENERAL CONSIDERATIONS; HETEROTIC MODELS. Genetics. 1964 01;49(1):49–67. Available from: https://doi.org/10.1093/genetics/49.1.49.
6. Zapata C, Álvarez G, Carollo C. Approximate variance of the standardized measure of gametic disequilibrium D'. American journal of human genetics. 1997;61 3:771–4. Available from: https://doi.org/10.1016/S0002-9297(07)64342-0.
7. Gabriel SB, Schaffner SF, Nguyen H, Moore JM, Roy J, Blumenstiel B, et al. The Structure of Haplotype Blocks in the Human Genome. Science. 2002;296(5576):2225–2229. Available from: https://doi.org/10.1126/science.1069424.
8. Phillips M, Kutch I, McHugh K, Taggard S, Burke M. Crossing design shapes patterns of genetic variation in synthetic recombinant populations of Saccharomyces cerevisiae. Scientific Reports. 2021 10;11:19551. Available from: https://doi.org/10.1038/s41598-021-99026-0.
9. Jónás Á, Taus T, Kosiol C, Schlötterer C, Futschik A. Estimating the effective population size from temporal allele frequency changes in experimental evolution. Genetics. 2016 Oct;204(2):723–735. Available from: https://doi.org/10.1534/genetics.116.191197.
